# Extended Synaptotagmin (ESyt) Triple Knock-Out Mice Are Viable and Fertile without Obvious Endoplasmic Reticulum Dysfunction

**DOI:** 10.1371/journal.pone.0158295

**Published:** 2016-06-27

**Authors:** Alessandra Sclip, Taulant Bacaj, Louise R. Giam, Thomas C. Südhof

**Affiliations:** 1 Department of Molecular and Cellular Physiology, Stanford University School of Medicine, 265 Campus Drive, Stanford, CA, 94305–5453, United States of America; 2 Howard Hughes Medical Institute, Stanford University Medical School, 265 Campus Drive, Stanford, CA, United States of America; Simon Fraser University, CANADA

## Abstract

Extended synaptotagmins (ESyts) are endoplasmic reticulum (ER) proteins composed of an N-terminal transmembrane region, a central SMP-domain, and five (ESyt1) or three C-terminal cytoplasmic C2-domains (ESyt2 and ESyt3). ESyts bind phospholipids in a Ca^2+^-dependent manner via their C2-domains, are localized to ER-plasma membrane contact sites, and may catalyze lipid exchange between the plasma membrane and the ER via their SMP-domains. However, the overall function of ESyts has remained enigmatic. Here, we generated triple constitutive and conditional knock-out mice that lack all three ESyt isoforms; in addition, we produced knock-in mice that express mutant ESyt1 or ESyt2 carrying inactivating substitutions in the Ca^2+^-binding sites of their C2A-domains. Strikingly, all ESyt mutant mice, even those lacking all ESyts, were apparently normal and survived and bred in a manner indistinguishable from control mice. ESyt mutant mice displayed no major changes in brain morphology or synaptic protein composition, and exhibited no large alterations in stress responses. Thus, in mice ESyts do not perform an essential role in basic cellular functions, suggesting that these highly conserved proteins may perform a specialized role that may manifest only during specific, as yet untested challenges.

## Introduction

Extended synaptotagmins (ESyts) are a family of endoplasmic reticulum (ER) proteins that are expressed in three isoforms, ESyt1, ESyt2, and ESyt3 [1]. Similar to conventional synaptotagmins, ESyts are composed of a short N-terminal extracytoplasmic sequence, a single transmembrane region (TMR), and multiple C-terminal C2-domains that partly bind to phospholipids in a Ca^2+^-dependent manner [1–3]. Different from synaptotagmins, however, ESyts contain a highly conserved central SMP-domain (for Synaptotagmin-like, Mitochondrial lipid binding Protein-domain) inserted between the TMR and the C2-domains, and 5 (ESyt1) or 3 C2-domains (ESyt2 and ESyt3) instead of the 2 canonical C2-domains of synaptotagmins. Because of their ER localization and their ability to bind to PI(4–5)P2 and other phospholipids in the plasma membrane, it has been suggested that ESyts may mediate formation of ER-plasma membrane contacts [3, 4]. Moreover, it was proposed that ESyts mediate lipid exchange between the ER and plasma membrane at these contacts because SMP-domains have a suggested role in lipid transfer between membranes [5–7]. Significant evidence for these conclusions was provided in yeast [8], but they have not yet been tested in higher eukaryotes using a genetic loss-of-function approach, and alternative functions for ESyts have also been proposed.

ER-plasma membrane contacts were first described in muscle [9], and later observed in a variety of other cell types, including neurons [4, 10, 11], yeast [8, 12, 13], and plants [14, 15]. ER-plasma membrane contacts have been associated with a variety of functions, such as transport of Ca^2+^ and lipids, control of ER shape and morphology, response to cellular stress, and organization of signaling networks [8, 16–20]. Several observations support the hypothesis that ESyts play a crucial role in the formation of ER-plasma membrane contacts. First, ESyts and tricalbins, which are the ESyt orthologs in yeast, directly localize in the cortical ER and are enriched in ER-plasma membrane contact sites [3, 4, 8]. Second, overexpression of ESyts induces formation of ER-plasma membrane contacts in HeLa and COS cells [3, 4]. Tethering of the ER to the plasma membrane in these experiments appeared to require interaction of the C2C- or C2A-domains of ESyts with PI(4–5)P2 phospholipids in the plasma membrane [2–4, 21]. Moreover, formation of ER-plasma membrane contact sites mediated by ESyt1 can be triggered by increases in cytoplasmic Ca^2+^, while ESyt2 and ESyt3 binding to the plasma membrane may occur at resting Ca^2+^-levels [4]. Third, triple knock-down of ESyts was shown to lead to a significant reduction of ER-plasma membrane contact sites in both resting and Ca^2+^-stimulated conditions [3], although these experiments did not eliminate expression of ESyts and the potential for off-target effects of knock-down experiments cannot be excluded.

Interestingly, double knock-out (KO) mice lacking ESyt2 and ESyt3 are viable, develop normally, and do not show any major phenotype [22]. The lack of phenotype in the ESyt2 and ESyt3 double KO mice could potentially be explained by compensation mediated by the remaining ESyt1, suggesting that the three ESyt isoforms may have redundant functions. To investigate this possibility and to gain a better understanding of ESyt functions, we generated triple KO mice lacking ESyt1, ESyt2 and ESyt3. These mice are viable and fertile, and exhibit no major alterations in brain structure, ER morphology, or synaptic protein composition. Moreover, loss of ESyts did not appear to increase susceptibility to stress stimuli or to induce a reorganization of the ER. These results suggest that ESyts are not essential for fundamental biological processes in mice, but do not rule out more specialized or redundant functions.

## Methods

### Generation of conditional and constitutive ESyt1, ESyt2, and ESyt3 KO mice, and of conditional and constitutive ESyt123 triple KO mice

**Fig 1:**
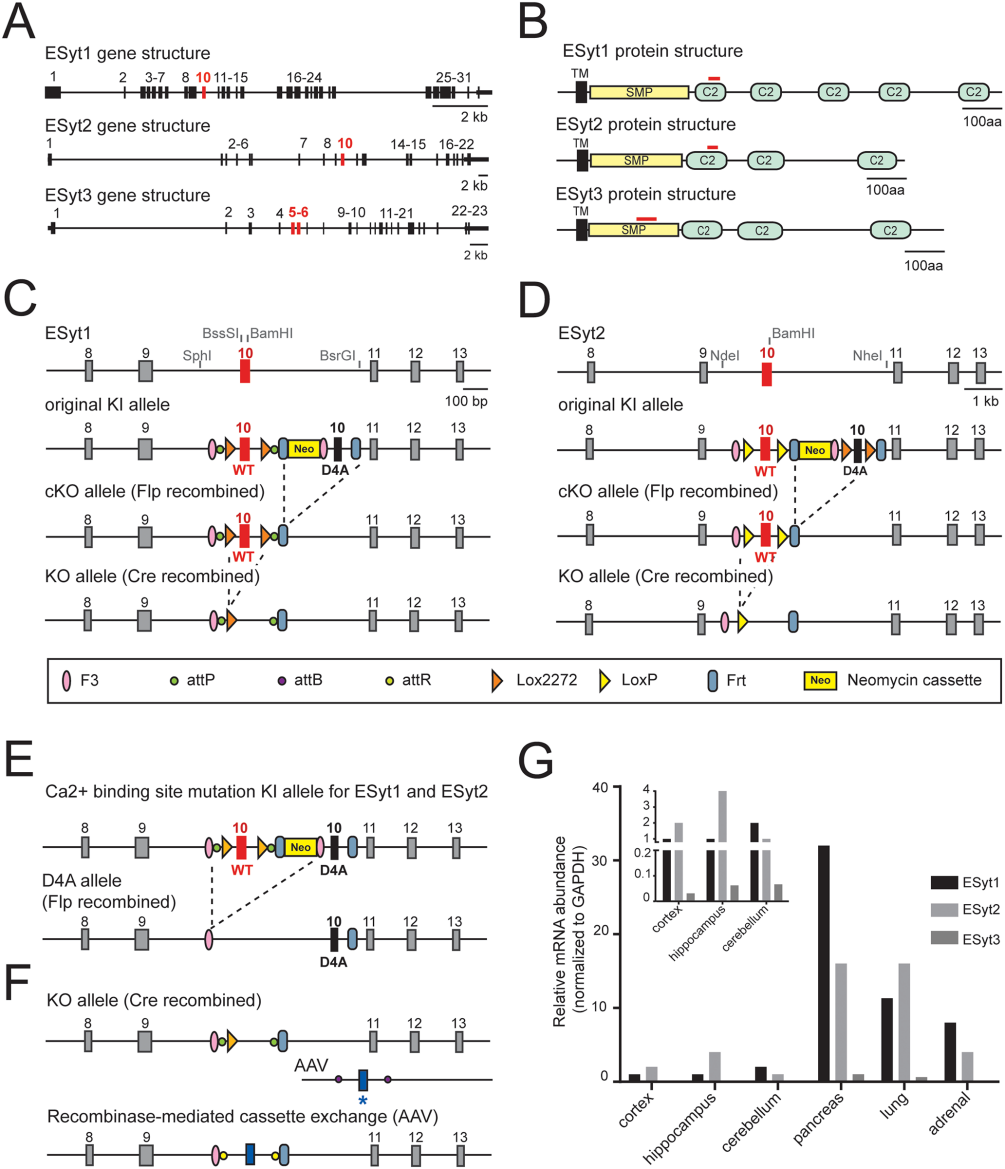
Generation of ESyt1, ESyt2 and ESyt3 triple KO mice. (A) ESyt1, ESyt2 and ESyt3 gene structures (exons are numbered). (B) ESyts domain organization (TM, transmembrane domain; SMP, synaptotagmin-like mitochondrial-lipid-binding protein). (C) Strategy used to generate conditional ESyt1 KO alleles. (D) Strategy used to generate conditional ESyt2 KO alleles. (E) Strategy for generating a knock-in line expressing mutantESyt1/ESyt2 unable to bind calcium in the C2A domain. Using Flp recombinase the WT exon 10 was removed in favor of expression of a mutated exon 10 (D4A), in which 4 Asp residues have been mutated into Ala to prevent calcium binding. (F) Strategy to additional modify the targeting vector by using recombinase-mediated cassette exchange method. This manipulation is possible only for ESyt1 mice. (G) Distribution of ESyts mRNA transcripts in different tissues of WT mice, measured by RT-PCR. In the insert, enlargement of the scale for ESyts mRNA level in the brain.

ESyt1 and ESyt2 conditional and constitutive KO mice were generated by homologous recombination in embryonic stem cells according to standard procedures, and subsequent breeding of the resulting mutant mice to mice expressing flp or cre recombinases in the germ line [23, 24]. Briefly, the targeting vectors were designed based on the strategy showed in Fig 1, and electroporated into embryonic stem cells. Homologously recombined clones were identified by Southern blotting, and used for blastocyst injection to produce chimeric mice carrying the floxed exon as well as a neomoycin selection cassette that was floxed. These mice were subsequently crossed to C57BL animals and their progeny was tested for germ-line transmission using PCR. The initial mouse lines (named 1EN and 2EN for ESyt1 and ESyt2 respectively) contained the neomycin cassette flanked by FRT sites. ESyt3 mice were obtained from the Eucomm consortium (ESyt3tm1a(EUCOMM)WTsi). This original line, called 3EN, contained the lacZ reporter and the neomycin resistance cassette flanked by FRT sites, as well as loxP sites that flank exons 5 and 6 for conditional or constitutive deletions. The sequences of the initial mutant alleles are provided in the SOMs.

To remove the neomycin resistance cassette, all three lines (1EN, 2EN and 3EN) were crossed to transgenic mice expressing FLP recombinase (Jackson Laboratory, RRID: IMSR_JAX:005703). The resulting mouse lines were named 1EF, 2EF and 3EF, and carried floxed exons, i.e. were conditional KOs, but lacked the neomycin resistance cassette. We crossed the three conditional KO lines to generate conditional ESyt123 triple KO mice (named 123EF). Mice were genotyped by PCR using a standard program: 95°C 2', (94°C 30'', 60°C 30'', 72°C 1' x 35 cycles), 72°C 7'. For genotyping we used the following primers: 1EN (TB12966) taccctccctttgtccctct, 1EN (TB12968) tagttgccagccatctgttg, 2EN (TB12972) agctggagagccagtgaatg, 2EN (TB12973) tcgccttcttgacgagttct, 3EN (TB13091) ttgatacccctgcacacctt, 3EN (TB13093) caagggcagtagagttccca, 1EF (TB13005) gacaggagatcgaggtggag, 1EF (TB13006) tgtgagtgcaggtgcctaag, 2EF (TB12971) tgagccatctgtcagtcagg, 2EF (TB12972) agctggagagccagtgaatg, 3EF (13091) ttgatacccctgcacacctt, 3EF (13092) tgctggaggtagaggtaggt. The expected bands are as follow:

1EN: WT, 1EF—no bands; 1EN—503 bp
2EN: WT, 2EF—no bands, 2EN—445 bp
3EN: WT, 3EF—no bands, 3EN—637 bp
1EF: WT—305, 1EF—435 bp
2EF: WT—245, 2EF—320 bp
3EF: WT—237, 3EF—409 bp

To generate constitutive ESyt123 triple KO mice (123EC), we crossed 123EF mice to a CMV-Cre mouse line (The Jackson Laboratory, RRID:IMSR_JAX:006054). Using this line, removal of LoxP-flanked exons occurs in all tissues, including germ cells [25]. Thus, this Cre line allowed for production of constitutive ESyt123 triple KO mice (123EC) after multiple generations of backcrossing. WT and constitutive KO mice were selected by PCR using the following primers: 1EC (TB12969) gttctgcagccgtgtcatag, 1EC (TB13006) tgtgagtgcaggtgcctaag, 2EC (TB12971) tgagccatctgtcagtcagg, 2EC (TB12974) cttctctgccaccctcagtc, 3EC (13091) ttgatacccctgcacacctt, 3EC (13094) tgcttgaattccagcttccc, 3EC (13092) tgctggaggtagaggtaggt. The expected band sizes are as follow:

1EC: WT—673, 1EC—491 bp
2EC: WT—979, 2EC—424 bp
3EC: WT—237, 3EC—491 bp

All procedures conformed to National Institutes of Health Guidelines for the Care and Use of Laboratory Animals and were approved by the Stanford University Administrative Panel on Laboratory Animal Care. Mice were maintained on a standard 12 hour light/dark cycle with food and water available ad libitum and their physical condition was checked regularly. All efforts were made to minimize the number of animals used and their suffering. Euthanasia of mice was performed by exposure to CO_2_ followed by cervical dislocation.

### Real time PCR

Total mRNA was extracted from mouse tissues using the PrepEase RNA Spin Kit (Affimetrix, Santa Clara, CA, USA) according to manufacturer instructions.ESyt1, ESyt2 and ESyt3 transcript levels were measured by qRT-PCR using the following predesigned FAM-dye coupled detection assays obtained from Integrated DNA Technologies (IDT, Coralville, IO, USA): Mm.PT.58.28544807, Mm.PT.58.30498250, Mm.PT.58.6713658, Mm.PT.58.42697478, Mm.PT.56a.32765675, Mm.PT.56a.7966789. Mouse GAPDH (4352932E, Applied Biosystems, Warrington, UK) was used as internal control. The qPCR assay was performed using the VeriQuest Probe One-Step qRT-PCR Master Mix (2X) (Affimetrix, Santa Clara, CA, USA).

### Survival analysis

**Fig 2:**
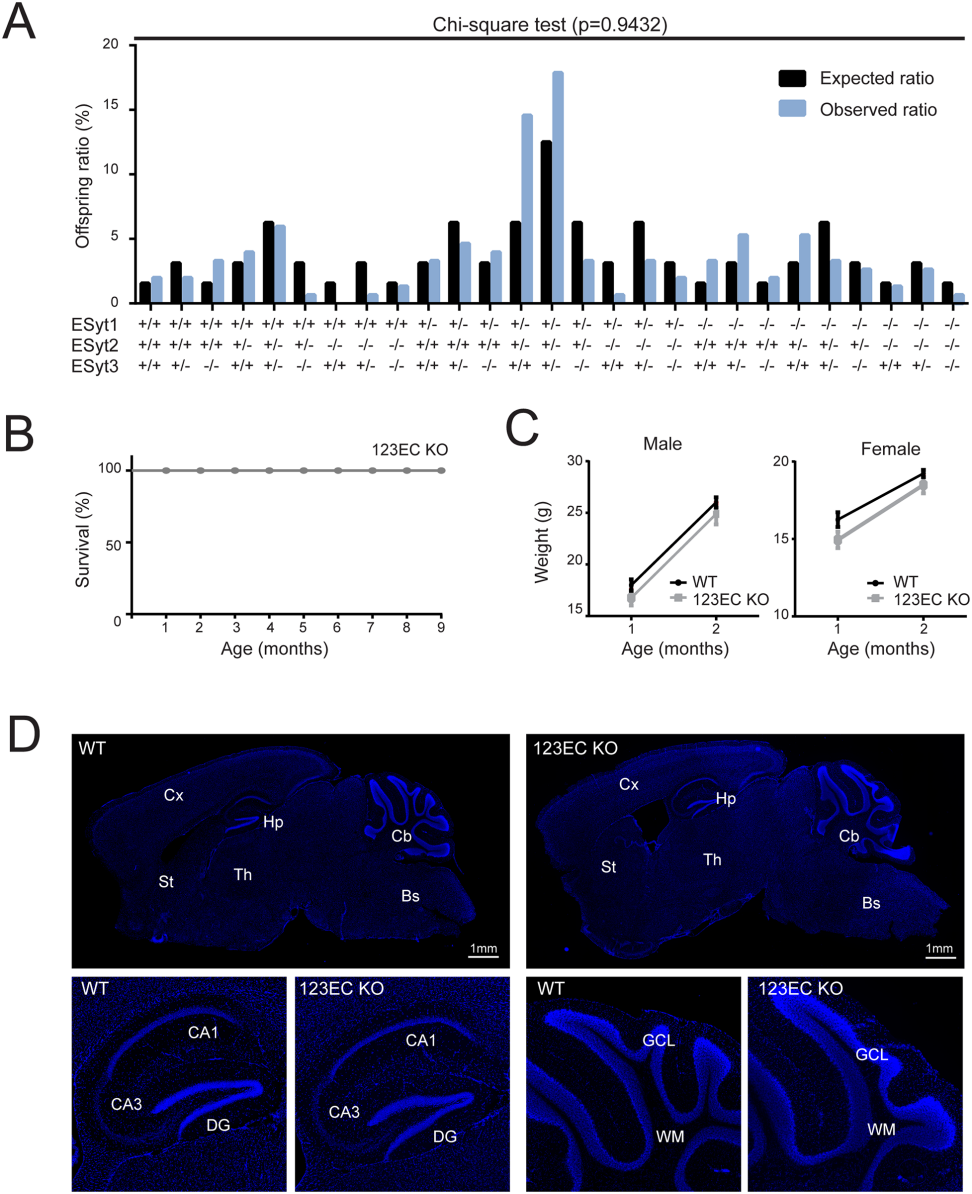
Constitutive ESyt123 triple KO mice are viable, fertile, develop apparently normally, and do not show major phenotypic changes. (A) Graph showing the distribution of the offspring obtained by crossing heterozygous 123EC KO mice. Black bars represent the expected ratio considering a Mendelian segregation of the alleles. Blue bars show the observed distribution. Chi-square test, p = 0.9432, n = 158. (B) Survival plot showing that homozygous 123EC KO mice are viable and have a normal lifespan (n = 8). (C) Both 123EC KO male and female mice do not show alterations in body weight if compared to WT mice. Data are shown as mean± SEM, One way ANOVA for repetitive measurements, p>0.05, n = 13 WT, n = 12 123EC. (D) Dapi staining of brain sections (top) confirming that 123EC KO mice do not show major alterations in the brain architecture. Magnification of the hippocampus (left) and cerebellum (right) confirming normal organizations of these structures in ESyt123 KO mice. Abbreviation: Cx, cortex; Hp, hippocampus; Cb, cerebellum; Bs, brainstem; St, striatum; Th, thalamus; DG, dentate gyrus; GCL, granule cell layer; WM, white matter.

Survival of constitutive ESyt123 triple KO mice was estimated by genotyping the offspring obtained from crosses between triple heterozygous 123EC mice. The genotypes were divided in 64 groups, as showed in Fig 2C and compared to frequency expected by Mendelian segregation of alleles.

### Brain sectioning and staining

Brains were fixed by intracardiac perfusion with PFA 4%. 30 μm sagittal sections were cut with a cryostat and mounted with mounting solution containing DAPI (0100–20, Southern biotech). Images were acquired with Olympus BX61VS fluorescence microscope.

### Immunoblotting

Cortical samples were dissected from both WT and 123EC homozygous mice, and homogenized in 0.32 M ice-cold sucrose buffer containing the following (in mM): 1 HEPES, 1 MgCl2, 1 EDTA, 1 NaHCO3 and 0.1 PMSF, at pH 7.4, in the presence of a complete set of protease inhibitors (Complete; Roche Diagnostics, Basel, Switzerland). Protein concentration was quantified using the Bradford Assay (Bio-Rad Protein Assay 500–0006, Munchen, Germany). 20–40 μg of brain homogenates were separated by SDS-PAGE using 4–20% mini protean TGX precast gels (Bio-Rad). Protein were transferred onto nitrocellulose membranes for 10 min at 2.5V using the Trans-blot turbo transfer system (Bio-Rad). Membranes were blocked in Tris-buffered saline (5% no-fat milk powder, 0.1% Tween20) for1h at room temperature. Primary antibodies were diluted in the same buffer and incubated overnight at 4°C.The following antibodies were used at 1:1000 dilution: ESyt1 (U6257), Esyt2 (U6253), syntaxin 1A (HPC-1), synaptobrevin 2 (69.1), SNAP-25 (71.1), MUNC-18 (K329), complexin 1–2 (L669), synaptotagmin 1 (41.1), synaptophysin (7.2), SV2 (U1129), synapsin (E028), liprin alpha (4396), Gpr78-BIP (ab21685, Abcam), HSP-27 (sc1048, Santa Cruz), HSP-70 constitutive (MA3-0066, Thermo Scientific), HSP-70 inducible (ADI-SPA-810-D, Enzo), VAP-A (NBP1-31237, Novus Biological), VAP-B (MAB58551-SP, Novus Biological), Nir2 (NBP1-69043, Novus Biological), Tubulin (T2200, Sigma), Actin (A1978, Sigma). Combinations of the following IRDye secondary antibodies were used at 1:10000 dilution: IRDye 800CW donkey anti mouse (926–32212), IRDye 680LT donkey anti mouse (926–68022), IRDye 800CW donkey anti rabbit (926–32213), IRDye 680LT donkey anti rabbit (926–68023), from LI-COR. Pseudo colors were then applied to the signals. Detection of the signal was obtained by Odyssey CLx imaging systems (LI-COR). Quantification was performed with Image Studio 5.2 free software.

### Lentiviral production

Lentiviruses were produced as described [26] in HEK293T cells by cotransfection with three helper plasmids (pRSV-REV, pMDLg/pRRE and vesicular stomatitis virus G protein expression vector) using calcium phosphate [27]. Lentiviruses were harvested with the medium 48 h after transfection, and snap-frozen in liquid N2. The following lentiviral constructs were used: 1. lentiviral vector (L309) expressing EGFP-Cre fusion protein and EGFP-deltaCre fusion protein, under the ubiquitin promoter; 2. lentiviral vector (L309) expressing mCherry-Cre fusion protein and mCherry-deltaCre fusion protein, under the synapsin promoter; 3. lentiviral vector (L309-gCaMP6M) expressing gCaMP6M under the control of synapsin promoter.

### Cortical and hippocampal neuronal cultures

P0 pups from 123EF homozygous mice were used to prepare hippocampal and cortical cultures as previously described [23]. Briefly, both hippocampal and cortical tissue was isolated using a dissection microscope, digested with papain for 20 min at 37°C, and then dissociated by gentle trituration. Neurons were plated onto round coverslips in 24 well plates or into 96 well plates. Plates and coverslip were pretreated with 2% matrigel (Collaborative Biomedical, Bedford, MA, USA) for 1 h at 37°C. 2mM AraC was added after 3 days in vitro (DIV). At DIV 4 cultures were treated with lentiviral vectors expressing EGFP-Cre fusion protein (referred as Cre) or EGFP-deltaCre fusion protein (referred as ΔCre), a deleted version of Cre which is unable to induce recombination (control condition) driven by an Ubiquitin promoter. Cells were used as DIV 14 for experiments. Expression of Cre or ΔCre was assessed by monitoring the expression of EGFP using a fluorescent microscope (Leica).

### Toxicity assays

Cortical neurons cultured into 96 well plates were treated at DIV 14 with the following drugs for 24h to induce neuronal death: DTT (0.5mM), Thapsigargin (5 μM), Tunicamycin (15 μM) and Paraquat (50 μM). After 24h neuronal death was assessed by MTT assay. Briefly, neurons were incubated with medium containing 0.4 mg of MTT (M5655, Sigma) for 4 h. After this incubation, the media was removed and formazol crystal were dissolved in HCl (1N): propanol 1:25. Neuronal survival was estimated by quantifying absorbance at 540 nm using a microplate absorbance reader (Apollo LB 912, Berthold Technologies GmbH & Co. KG). Three independent cultures were used for the experiment.

### Analysis of ER distribution

Hippocampal neurons were transfected at DIV10 with pCMV5-Sec61β-EGFP and L309-mCherry constructs using the calcium phosphate method. At DIV 14 neurons were fixed in 4% PFA, and imaged using a confocal microscope (A1RSi+, Nikon) controlled by the NIS-Elements AR software (Nikon). All acquisition parameters were kept constant between conditions. Three independent cultures were used for the experiment.

### Ca^2+^ imaging experiments

Ca^2+^ imaging experiments were performed on hippocampal neurons from 123EF homozygous mice, plated on black-walled clear flat-bottomed 96-well plates (Costar 3603). At DIV4 neurons were infected with a lentivirus expressing GCaMP6M, in combination with lentivirus expressing either mCherry-Cre or mCherry-ΔCre, to produce knockout or WT cultures, respectively. Ca^2+^ imaging was performed on neurons at DIV14. Calcium activity recordings were performed on a Molecular Devices ImageXpress Micro inverted epifluorescence microscope in the Stanford High-Throughput Bioscience Center facility using UV excitation, a 10X 0.3 Plan Fluor objective, and the FITC-FIXED (525 ± 30 nm) and TRITC-FIXED (607 ± 36 nm) filters. First, one TRITC in-focus image was taken and then in the FITC channel, a 5-minute recording was made (300 images taken at 1 frame/sec) in a controlled environment of 37°C and 5% CO2. Image data were analyzed using the MetaXpress software using built-in functions to threshold the TRITC image and identify cell bodies (mCherry-Cre, localized in the nucleus), create regions of interest (ROIs) around the cell bodies, overlay those ROIs on the FITC time-lapse images, and export the ROI FITC intensities as a function of time into a text file. The text file was processed using some custom Matlab scripts.

## Results

### Generation of conditional ESyt123 triple KO mice

The ESyt family comprises three evolutionary conserved, closely related proteins: ESyt1, ESyt2 and ESyt3. In mice, the ESyt1, ESyt2 and ESyt3 genes are located on chromosomes 10, 12 and 9, respectively, and are composed of 31, 22 and 23 exons (Fig 1A). The ESyt genes encode for membrane proteins containing a single N-terminal TMR, a conserved central SMP-domain, and either five (E-Syt1) or three C-terminal C2-domains (E-Syt2 and E-Syt3; Fig 1B).

To enable analysis of the functions of ESyts, we generated conditional ESyt123 triple KO mice by making conditional ESyt1, ESyt2, and ESyt3 KO mice, and crossed these mice with each other. ESyt3 mice were obtained from Eucomm consortium. In these mice, exons 5–6 (display in red in Fig 1A) were targeted, so that cre recombination removes these exons and causes insertion of a premature stop codon, therefore creating a conditional null allele.

ESyt1 and ESyt2 conditional mutant mice were generated by a dual purpose targeting strategy that allows generating two independent mouse lines from the same homologous recombination reaction as outlined in Fig 1C. This strategy depends on inserting two different pairs of frt recombination sites, such that random recombination via one or the other pair produces either conditional KO mice in which a wild-type exon 10, which encodes part of the C2A-domain in both ESyt1 and ESyt2 (Fig 1B), is flanked by loxP sites, or in which a mutant exon 10 with inactivating substitutions in the Ca^2+^-binding sites of its C2A-domain replaces the wild-type exon 10.

For Esyt1, specifically, the targeting vector contained in intron 9 a first F3 frt-recombination site, an AttB site, and a 5’ Lox2272 site followed by the wild-type exon 10. The first copy of the following intron 10 then contained a second Lox2272 site (thus rendering exon 10 flanked by loxP sites) as well as a second AttP site, followed by a standard Frt-recombination site, a neomycin resistance cassette, and a second F3 site. After this cassette, a second, mutant copy of exon 10 was inserted. In the mutant exon 10, the four aspartate residues responsible for Ca^2+^-binding were replaced by alanine residues (D4A), and the mutant exon was followed by a second Frt site (Fig 1C). As a result, flp-recombinase mediated excision of the sequences flanked by the classical frt sites produces a conditional KO allele with exon 10 flanked by loxP sites, whereas flp-recombinase mediated excition of the sequences flanked by F3 sites produced a constitutive mutant allele in which the C2A-domain Ca^2+^-binding sites of the C2A-domain are selectively abolished.

For Esyt2, the targeting vector was identical to that for ESyt1, except that the second mutant copy of exon 10 was additionally flanked by loxP sites, and no AttB sites were included (Fig 1D). Thus, the ESyt2 mutant allele not only enables generation of the same type of conditional KO mice and constitutively mutant mice as the ESyt1 mutant allele, but also allows conditional deletion of the mutant ESyt2 protein.

Once we had obtained mice with the originally recombined mutant ESyt1 and ESyt2 alleles using homologous recombination in ES cells and generation of mice from these ES cells, we crossed the original mutant mice with transgenic mice expressing FLP recombinase driven by the β-actin (ACTB) promoter [28]. Offspring from these crosses were predominantly recombined via the standard frt sites that were much preferred by the transgenic FLP recombinase, but a low incidence of recombination via the F3 sites was also observed, allowing us to obtain both conditional KO (cKO) mice for ESyt1 and ESyt2 as well as constitutive knock-in (KI) mice for these genes in which the C2A-domain Ca^2+^-binding sites were mutated (Fig 1C–1E). In the cKO mice, Cre recombination will lead to deletion of exon 10, causing a frameshift in the protein and insertion of an early stop codon. Moreover, in the ESyt1 line, the inserted AttB sites can be used to manipulate exon 10 by introducing different mutations using AAV vectors and the recombinase mediated cassette exchange method (Fig 1F).

After their generation, we crossed Esyt1, Esyt2 and Esyt3 cKO mice with transgenic mice expressing Cre under the CMV promoter, therefore allowing recombination in the germline and generation of a constitutive KO mice (S1A Fig). Finally, we produced constitutive ESyt123 triple KO mice (123EC) as well as ESyt123 triple cKO mice by crossing the single KO and cKO mice with each other in a multi-generation breeding scheme.

To test if the targeting strategy was effective in deleting the ESyt isoforms, we measured the expression levels forESyt1, ESyt2 and ESyt3 in the cortex and lung of ESyt123 triple KO and wild-type (WT) mice by RT-PCR and immunoblotting. We found that the mRNA levels of ESyt1, ESyt2 and ESyt3 were significantly lower in constitutive KO mice compared to WT littermates (S1B Fig). ESyt mRNAs, however, were not completely ablated, probably because the mRNAs are decreased by nonsense-mediated decay which operates for some but not for all mRNA species, and not all frameshift mutations in a gene lead to a loss of its corresponding mRNA. ESyt1 and ESyt2 proteins, however, were not detected in the KO mice, confirming that the proteins were being removed (S1C Fig).

### ESyts are not essential for mouse survival and fertility

Surprisingly, constitutive ESyt123 triple KO mice are viable. After systematic evaluation of the offspring obtained from breedings of constitutive ESyt1^+/-^ESyt2^+/-^ESyt3^+/-^ mice, we observed that the allelic distribution of the offspring followed a Mendelian pattern of inheritance (Fig 2A). The offspring ratio for the different genotypes was in fact comparable to the expected ratio, although some skewing towards the ESyt1^+/-^ESyt2^+/-^ESyt3^+/+^ and ESyt1^+/-^ESyt2^+/-^ESyt3^+/-^was observed (Fig 2A). Constitutive ESyt123 triple KO mice displayed a normal lifespan as far tested, as no premature death was observed in the colony (Fig 2B). All mice were fertile and did not show obviously altered spontaneous behaviours compared to WT mice. Moreover, both male and female triple KO mice had a normal size and weight as compared to their WT littermates (Fig 2C), and did not present any obvious morphological defects. Finally, no obvious structural changes in the anatomy of the brain were observed, as shown from dapi staining of sagittal brain slices from 3 months old WT and ESyt123 constitutive triple KO mice (Fig 2D). Therefore we conclude that ESyts are not essential for mouse survival, viability, and fertility.

### Loss of ESyt123 does not detectably alter the brain protein composition

**Fig 3:**
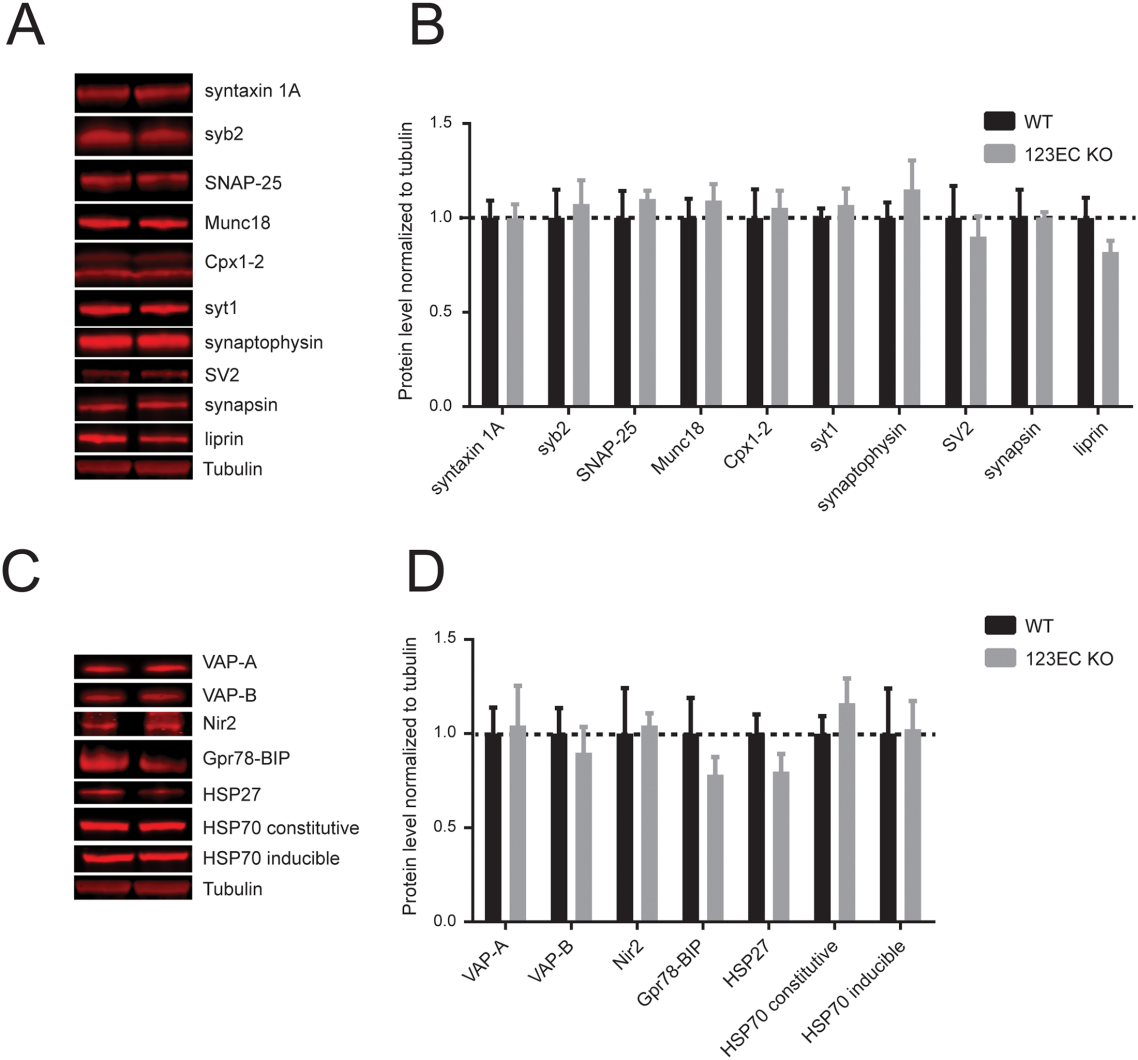
Loss of ESyts does not affect the level of synaptic and ER markers in the brain. (A and B) Immunoblots and relative quantifications of major pre-synaptic proteins in WT and constitutive ESyt123 triple KO mice. Data are shown as means ± SEM, Student’s t-test, p>0.05, n = 4. (C and D) Western blot and relative quantification of ER proteins, showing no differences between WT and 123EC KO mice. Data are shown as means ± SEM, Student’s t-test, p>0.05, n = 4.

To examine whether deletion of ESyts leads to an alteration of synaptic proteins in the brain, we performed immunoblotting analyses of cortical tissue dissected from 3 months old WT and constitutive ESyt123 triple KO mice. The levels of major presynaptic proteins, such as SNARE proteins (syntaxin-1, synaptobrevin-2, SNAP-25), Ca^2+^-sensor proteins (synaptotagmin1), vesicular proteins (SV2, synapsin, synaptophysin), and active zone proteins (α-liprins) remained unchanged in ESyt123 KO versus WT control samples (Fig 3A and 3B). These data suggest that loss of ESyts does not affect the protein composition of synaptic sites, and therefore the biochemical integrity of the brain was maintained. Moreover, the levels of proteins localized at ER-PM contacts, such as VAP-A, VAP-B, and Nir2, as well as proteins associated to ER stress, such as Gpr78-BIP, HSP-27, HSP-70, also did not exhibit significant differences in ESyt123 triple KO brains as compared to WT controls (Fig 3C and 3D).

### Loss of ESyt123 does not impair neuronal survival in response to stress

**Fig 4:**
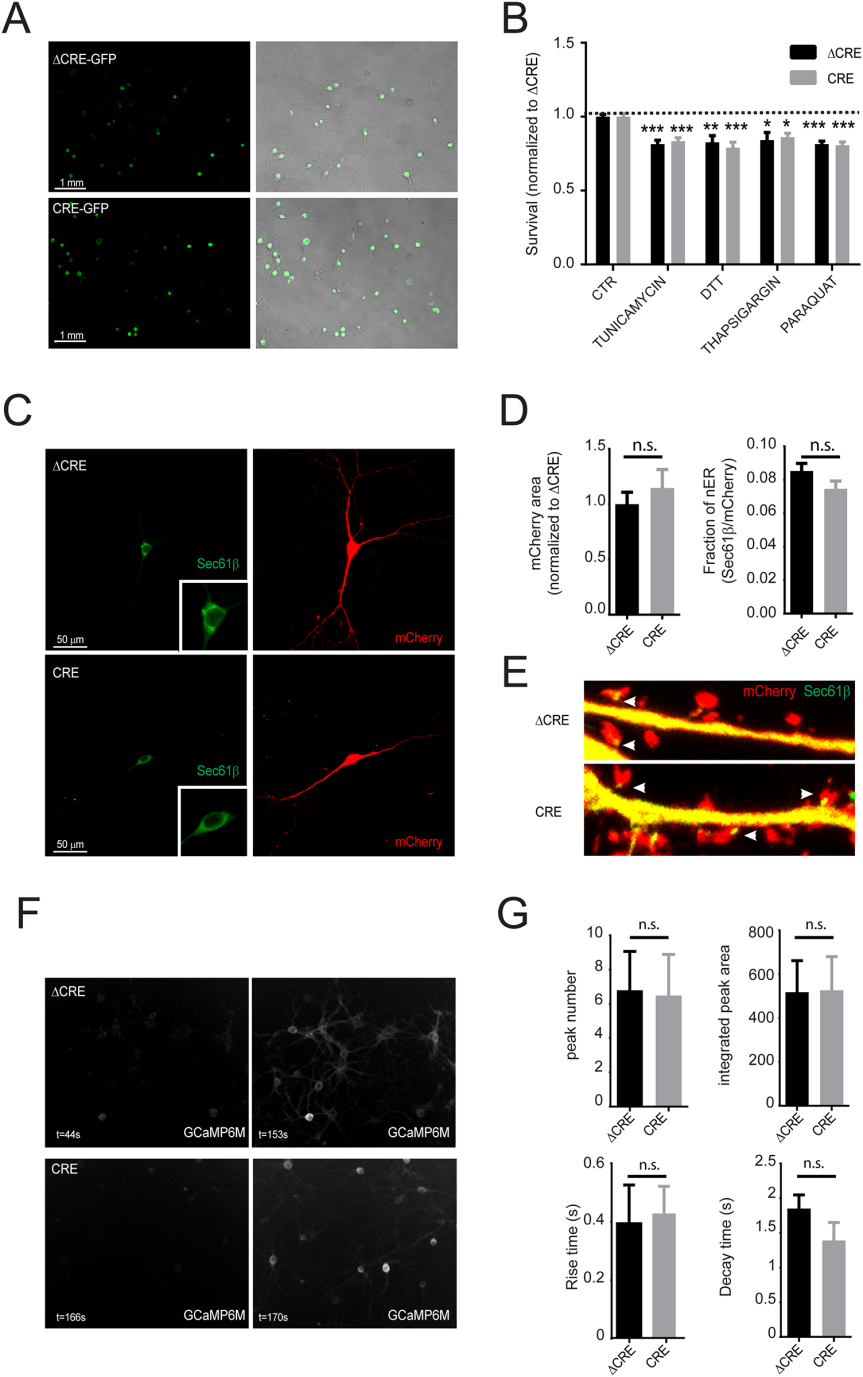
Triple ESyt123 KO does not increase the susceptibility of neurons to stress, induce obvious changes in the ER, or affect calcium dynamics in neurons. (A) Hippocampal cultures obtained from ESyt123 conditional KO mice and infected with lentiviruses expressing GFP-Cre and GFP-ΔCre. Images show GFP fluorescence merged with brightfield signal to illustrate the high efficiency of infection of the virus for both Cre and ΔCre conditions. (B) Neuronal cell death in response to mild stress (DTT, Tunicamycin, Thapsigargin and Paraquat) was assessed by the MTT assay. The stress conditions induced partial neuronal cell death, which was unchanged in neurons infected with Cre or ΔCre, suggesting that loss of ESyts does not increase susceptibility to toxic agents. Data were normalized to the control ΔCre condition, and are expressed as means ± SEM. Two-way ANOVA, Bonferroni post-hoc test, *p<0.05 vs CTR, **p<0.01 vs CTR, ***p<0.001 vs control, n = 8. (C) Confocal images showing hippocampal neurons transfected with Sec61β-EGFP in green and mCherry signal in red. Insert shows a magnification of the soma to better visualize the ER associated with the nucleus. (D) Quantification of the total neuronal area (mCherry signal) and the fraction of the nuclear-associated ER (Sec61β area normalized to mCherry area) showing no difference between ΔCre and Cre treated neurons. Data are expressed as means ± SEM. Student t-test, p>0.05, n = 18 ΔCre, n = 15 Cre. (E) Representative images of Sec61β-EGFP signal (green) acquired at higher exposure, showing that cytoplasmic ER is present in dendrites as well as in the neck of a subset of spines (see arrowheads) in both ΔCre and Cre treated neurons. The mCherry signal (red) allows visualization of dendrites and dendritic spines. (F) Representative images of GCaMP6M-expressing hippocampal neurons at resting (left panel-absence of Ca2+-signal) or during network activity (right panel-presence of a Ca2+-signal) for ΔCre (top panel) and Cre (low panel) treated neurons. Calcium activity is present in both ΔCre and Cre treated neurons. (G) Quantification of the number of calcium peaks, the integrated peak area, as well as the rise time and decay time kinetics, during 5min recording of neuronal activity. No significant differences has been observed between ΔCre and Cre treated neurons. Data are expressed as means ± SEM. Student t-test, p>0.05, n = 5 pups (total of analyzed neurons = 184) ΔCre, n = 5 pups (total of analyzed neurons = 129) Cre.

It has been suggested that ER-plasma membrane sites control stress responses [8, 16, 29]. In yeast, cells lacking ER-plasma membrane contacts are hypersensitive to ER stress induced by tunicamycin, and display alterations in the unfolded protein response [8]. To test the hypothesis that ESyts support neuronal survival after exposure to stress stimuli by maintaining the integrity of ER and regulating ER chaperone proteins, we performed toxicity assays. We cultured cortical neurons from conditional ESyt123 triple cKO mice, and infected them with lentiviruses expressing EGFP-tagged wild-type Cre-recombinase (test, to delete ESyts) or mutant Cre-recombinase (ΔCre, as a control). As shown in Fig 4A, infection of the cultures with lentiviruses was efficient, resulting in the expression of the EGFP-tagged proteins in all neurons. At DIV14, we exposed the neurons to different pharmacological agents that induce ER and/or mitochondrial stress (DTT, thapsigargin, tunicamycin, paraquat). Concentrations that induced mild toxicity were used in all cases. As shown in Fig 4B, all toxic stimuli induced 20–30% neuronal death. However, no differences in the toxicity were observed in Cre- vs ΔCre-expressing neurons, suggesting that ESyt123 KO cells are not hypersensitive to toxic agents and ER stress.

### ESyt123 loss does not alter ER morphology in neurons

To further elucidate if removal of ESyts leads to alterations in ER morphology, we transfected hippocampal neurons at DIV10 with a plasmid expressing full length Sec61β tagged with EGFP under the control of the CMV promoter. In addition, we co-transfected the cells with a plasmid expressing soluble mCherry to visualize the morphology of the neurons (Fig 4C, 4D and 4E). To investigate if the ESyt triple KO affected neuronal morphology, we first quantify the area filled by mCherry. We found no differences between WT and KO neurons, suggesting that loss of ESyts does not affect dendritic outgrowth or neuronal morphology (Fig 4D). We further analyzed the fraction of nuclear ER in these neurons by normalizing the area of the EGFP-Sec61β signal to the mCherry signal. Also in this case, we did not observed differences between the groups, suggesting that ESyts are not required for shaping ER morphology (Fig 4D). Finally, we checked if cortical ER was altered in ESyt KO neurons. In neurons, ER can be detected in dendrites [11, 30–32], in the head or neck of a subset of dendritic spines, where is known as spine apparatus [10, 11, 33–35], as well as in axons [36–41]. We found that the localization of EGFP-Sec61β signal was maintained in dendrites as well as in dendritic spines (Fig 4E), supporting the idea that removal of ESyts does not massively alter ER morphology.

### Removal of ESyt123 does not alter single cell calcium dynamics during network activity in vitro

ER-plasma membrane contacts have been described as sites for calcium regulation [3, 8, 42]. Therefore it has been suggested that changes in density, size and distance between ER-plasma membrane contacts can exert profound effect on calcium dependent biological functions [19]. To assess the effect of ESyts in the regulation of calcium dynamics, we monitor activity dependent calcium transient in populations of hippocampal neurons infected with the calcium indicator GCaMP6M [43] in response to spontaneous network activity (Fig 4F and 4G). As shown in Fig 4G, we did not observed any significant differences in the number of calcium peaks, in the integrated peak area, as well as in the rise time and decay time in control (infected with ΔCRE) and Esyt123 knockout neurons (infected with Cre). These results suggest that loss of ESyts in hippocampal neurons does not affect the calcium dynamics in response to neuronal activity.

## Discussion

ESyts were identified a decade ago as a family of homologous Ca^2+^-binding proteins with similarity to synaptotagmins [1], but their functions and importance have remained enigmatic despite intense study. ESyts are highly conserved in evolution, and are expressed from multiple homologous genes in nearly all eukaryotic cells, including mammals, yeast, and plants. Pioneering work in yeast suggested that tricalbins, the yeast homologs of ESyts, may be important for organizing ER-plasma membrane contacts and enabling lipid transport at these contacts [8], a hypothesis that was strongly supported by work in mammalian tissue culture cells [3]. However, other reports suggested alternative functions [16, 17, 19, 44]. To assess whether ESyts are indeed important for fundamental cellular processes and to enable studies in particular of their Ca^2+^-dependent functions, we here generated a series of mutant mice that allow assessing their essential roles. Strikingly, we found that ESyts perform no required function in the basic biological activities of a mouse, suggesting that their functions are either redundant, subtle, or specific to a unique and unexpected biological context. Regrettably, our data do not reveal what ESyts do physiologically, except to allow the conclusion that their functions are not of fundamental significance for mouse survival and fertility, and that thus ESyts do not perform an essential role in the basic biology of mouse cells.

Recently, a series of reports have implicated ESyts in tethering ER to the plasma membrane, and have suggested that ESyts may have a role in the regulation of lipid transport, ER morphology, and calcium dynamics, as well as in stress responses and signal transduction [8, 16–20, 44]. These reports implied that ESyts may have important functions in the brain; since ER-plasma membrane contacts have been observed frequently in axons and dendrites [4, 10, 11, 30–32, 45–47], and suggested to contribute to neuronal protein trafficking, neuronal morphogenesis, and synaptic function [44]. However, no systematic analysis of ESyt functions in KO models tested these ideas. To get a better understanding of ESyts function in a complex organism, we generated a triple KO mice in which all three isoforms of ESyts had been deleted (ESyt1, ESyt2, and ESyt3). These mice were viable and developed normally. Moreover, ESyts seemed to be unnecessary for the regulation of brain development and for the control of neuronal morphology or function. In fact, we found that the anatomical architecture of the brain was normal in ESyts KO mice, and the dendritic arborisation and spine formation, as well as calcium dynamics and firing properties, were maintained in cultured hippocampal neurons lacking ESyts. Moreover, we observed that the expression levels of major synaptic proteins were unchanged in the brain of ESyt123 triple KO mice, suggesting that the biochemical integrity of the brain was not impaired after removal of ESyts. Finally, ESyt-deficient mice were fully able to perform basic behaviours, such as feeding and breeding, suggesting that their brains are not fundamentally impaired. Similar observations were made in double KO mice for ESyt2 and ESyt3 [22], confirming that the lack of phenotype reported by the authors was not due to compensation of the remaining ESyt1.

The negative results that we obtained can potentially be explained by the hypothesis that in complex systems, other tethering factors may control the formation of ER-plasma membrane sites, and compensate for absence of ESyts [8, 22]. In fact, it has been shown in yeast that removal of tricalbins (orthologs to the ESyt family) is not sufficient to disrupt ER-plasma membrane contact sites [8]. In contrast, removal of three other proteins in addition to tricalbins is required to completely abolish ER-plasma membrane tethers: Ist1 (a member of the TMEM16 ion channel family), and Scs2 and Scs22 (orthologs to the vesicle associated membrane protein associated protein, VAP) [8]. Loss of ER-plasma membrane contacts using this manipulation leads to reorganization of the ER, with enlargement of the nuclear ER and complete loss of cytoplasmic ER, to accumulation of PI4P in the plasma membrane, and increases in the unfolded protein response as well as hypersensitivity to stress stimuli. In support of this hypothesis, we also observed that removal of ESyts was unable to induce reorganization of the ER network in neurons, and failed to increase the susceptibility of neurons to stress.

Despite the localization of ESyts on the ER and their ability to bind phosphoinositides and other negatively charged phospholipids that are resident in the plasma membrane, their accumulation on ER-plasma membrane contacts in response to increase in cytosolic Ca^2+^, and the increased in ER-plasma membrane contact induced by their overexpression support the idea that ESyts exert some function in the cytoplasmic ER [3, 4, 48]. Our data demonstrate that their contribution is not essential, however, suggesting that other proteins with redundant functions may compensate for their absence or that the functions of ESyts are generally dispensable. Understanding the role of cytoplasmic ER and ER-plasma membrane contact sites in eukaryotic cells and in neurons thus remains an open challenge.

## Supporting Information

S1 Fig (A) Schematic of the breeding strategy used to obtain constitutive Esyt123 triple KO mice starting with the conditional KO mouse lines. ESyt123 triple cKO females were crossed with CMV-CRE males to generate constitutive Esyt123 triple KO mice after further interbreeding. (B) RT-PCR measurements of ESyt1, ESyt2 and ESyt3 mRNA levels in the cortex and lung of WT and ESyt123 triple KO mice (123EC KO). Levels were normalized to GAPDH. Data are shown as means ± SEM, n = 3. Note that mRNA measurements are not suitable for assessing the efficacy of a conditional KO since for many mRNAs, nonsense-mediated decay that destroys mRNAs containing a disrupted open reading frame either does not operate at all or is inefficient. Thus, many null alleles exhibit normal or partial mRNA levels that, however, do not encode a protein. (C) Representative immunoblots for ESyt1 and ESyt2 showing that these proteins are not detectable in brain and lung samples from constitutive ESyt123 triple KO mice.
(DOC)
