# Correction: Extended Synaptotagmin (ESyt) Triple Knock-Out Mice Are Viable and Fertile without Obvious Endoplasmic Reticulum Dysfunction

**DOI:** 10.1371/journal.pone.0298645

**Published:** 2024-02-06

**Authors:** Alessandra Sclip, Taulant Bacaj, Louise R. Giam, Thomas C. Südhof

With this notice we provide amended versions of Fig 3, S1 Fig, and the Data Availability statement in [1].

Fig 3 has been updated to address the following questions:

- Concerns were raised that the Munc18 and Syt1 panels of Fig 3A appeared similar and did not appear to match the primary image data for these experiments. In the updated figure, these panels have been replaced with different data from the original experiments that look more dissimilar.
- In reviewing the primary data for Fig 3C it came to light that HSP70 inducible blots had been displayed in the HSP70 constitutive panel. This error has been corrected in the updated figure.
- The Tubulin control panels in Fig 3A and 3C were replaced to correct a loading order discrepancy: review of the primary data revealed that the loading order for Tubulin panels in the original figure differed from the loading order in other panels of Fig 3A and 3C.
- VAP-A and VAP-B panels were replaced in the updated Fig 3C with a better selection, although no issues were raised about these panels in the published figure.

In addition, concerns were raised about similarities between the Tubulin panels in Fig 3A and 3C and the Actin panel for Cortex (Cx) experiments in S1C Fig. The updated Fig 3 legend clarifies that Tubulin data are intentionally reused in Fig 3A and 3C as the data for these two panels were obtained in the same set of experiments. S1C Fig has been revised to report the correct loading control data for each blot experiment.

These figure updates do not impact the results and conclusions as stated in the article [1]. The original images underlying Fig 3A, Fig 3C, and S1C Fig are provided in S1–S3 Files.

In addition, the Data Availability statement for this article is updated to:

Underlying data for images in amended versions of Fig 3 and S1 Fig are in supporting information files with the accompanying Correction and at https://purl.stanford.edu/vq040hz0549.

**Fig 3:**
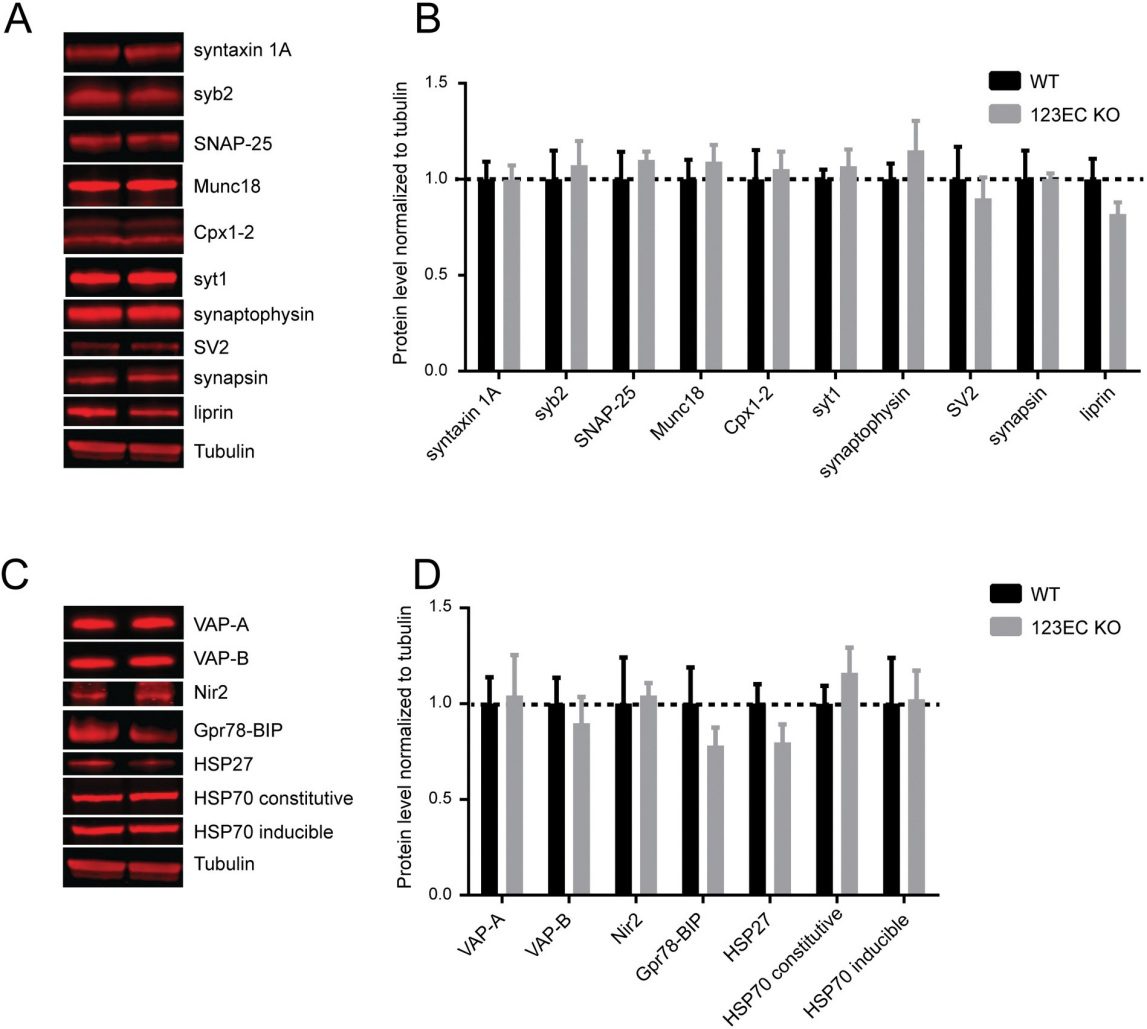
Loss of ESyts does not affect the level of synaptic and ER markers in the brain. A and B) Immunoblots and relative quantifications of major presynaptic proteins in WT (lane 1) and constitutive Esyt123 triple KO mice (lane 2). Data are shown as means ± SEM, Student’s t-test, p>0.05, n = 4. (C and D) Western blot and relative quantification**s** of ER proteins reveal no significant differences between WT (lane 1) and 123EC KO (lane 2) mice. Data are shown as means ± SEM, Student’s t-test, p>0.05, n = 4. The same Tubulin data are shown in Fig 3A and 3C because experiments for these panels were performed in parallel with the same protein samples and loaded amounts of protein. Tubulin controls were run for gel 1 (probing for SV2, Syntaxin 1, Complexins, HSP70), gel 2 (probing for Liprins, Synapsins, SNAP25, HSP70), and gel 4 (probing for Vapb, and Vap-a). Gels 3 (Munc18, Syt1, Synaptophysin, Syb2) and 5 (Grp78) were not probed for tubulin, but were run in parallel with gel 1, 2, and 4. Original full-length blots are deposited at https://purl.stanford.edu/vq040hz0549.

## Supporting information

S1 Fig (A) Schematic of the breeding strategy used to obtain constitutive Esyt123 triple KO mice starting with the conditional KO mouse lines. Esyt123 triple cKO females were crossed with CMV-CRE males to generate constitutive Esyt123 triple KO mice after further interbreeding. (B) RT-PCR measurements of Esyt1, Esyt2 and Esyt3 mRNA levels in the cortex and lung of WT and Esyt123 triple KO mice (123EC KO). Levels were normalized to GAPDH. Data are shown as means ± SEM, n = 3. Note that mRNA measurements are not suitable for assessing the efficacy of a conditional KO since for many mRNAs, nonsense-mediated decay that destroys mRNAs containing a disrupted open reading frame either does not operate at all or is inefficient. Thus, many null alleles exhibit normal or partial mRNA levels that, however, do not encode a protein. (C) Representative immunoblots for Esyt1 and Esyt2 showing that these proteins are not detectable in brain and lung samples from constitutive Esyt123 triple KO mice (loading controls = actin).
(TIF)

S1 File Original images underlying Fig 3A. Annotated and individual unannotated images underlying all western blots in this figure panel.
(PDF)

S2 File Original images underlying Fig 3C. Annotated and individual unannotated images underlying all western blots in this figure panel except for Nir2. The white box is incorrectly positioned on the Gpr78 image: lanes 5–6 are shown in the figure.
(PDF)

S3 File Original images underlying S1C. Annotated and individual unannotated images underlying western blots.
(PDF)
