# Supplementary material for: Extended Synaptotagmin (ESyt) Triple Knock-Out Mice Are Viable and Fertile without Obvious Endoplasmic Reticulum Dysfunction
Source: PLoS One. 2016 Jun 27;11(6):e0158295. doi: 10.1371/journal.pone.0158295 (PMC4922586; doi:10.1371/journal.pone.0158295)
Supplement: S1 Fig — (A) Schematic of the breeding strategy used to obtain constitutive Esyt123 triple KO mice starting with the conditional KO mouse lines. ESyt123 triple cKO females were crossed with CMV-CRE males to generate constitutive Esyt123 triple KO mice after further interbreeding. (B) RT-PCR measurements of ESyt1, ESyt2 and ESyt3 mRNA levels in the cortex and lung of WT and ESyt123 triple KO mice (123EC KO). Levels were normalized to GAPDH. Data are shown as means ± SEM, n = 3. Note that mRNA measurements are not suitable for assessing the efficacy of a conditional KO since for many mRNAs, nonsense-mediated decay that destroys mRNAs containing a disrupted open reading frame either does not operate at all or is inefficient. Thus, many null alleles exhibit normal or partial mRNA levels that, however, do not encode a protein. (C) Representative immunoblots for ESyt1 and ESyt2 showing that these proteins are not detectable in brain and lung samples from constitutive ESyt123 triple KO mice. (DOC) [file pone.0158295.s001.doc]

SUPPLEMENTARY MATERIAL for A. Sclip et al.,

“Extended synaptotagmin (ESyt) triple knockout mice are viable and fertile without obvious endoplasmic reticulum dysfunction”


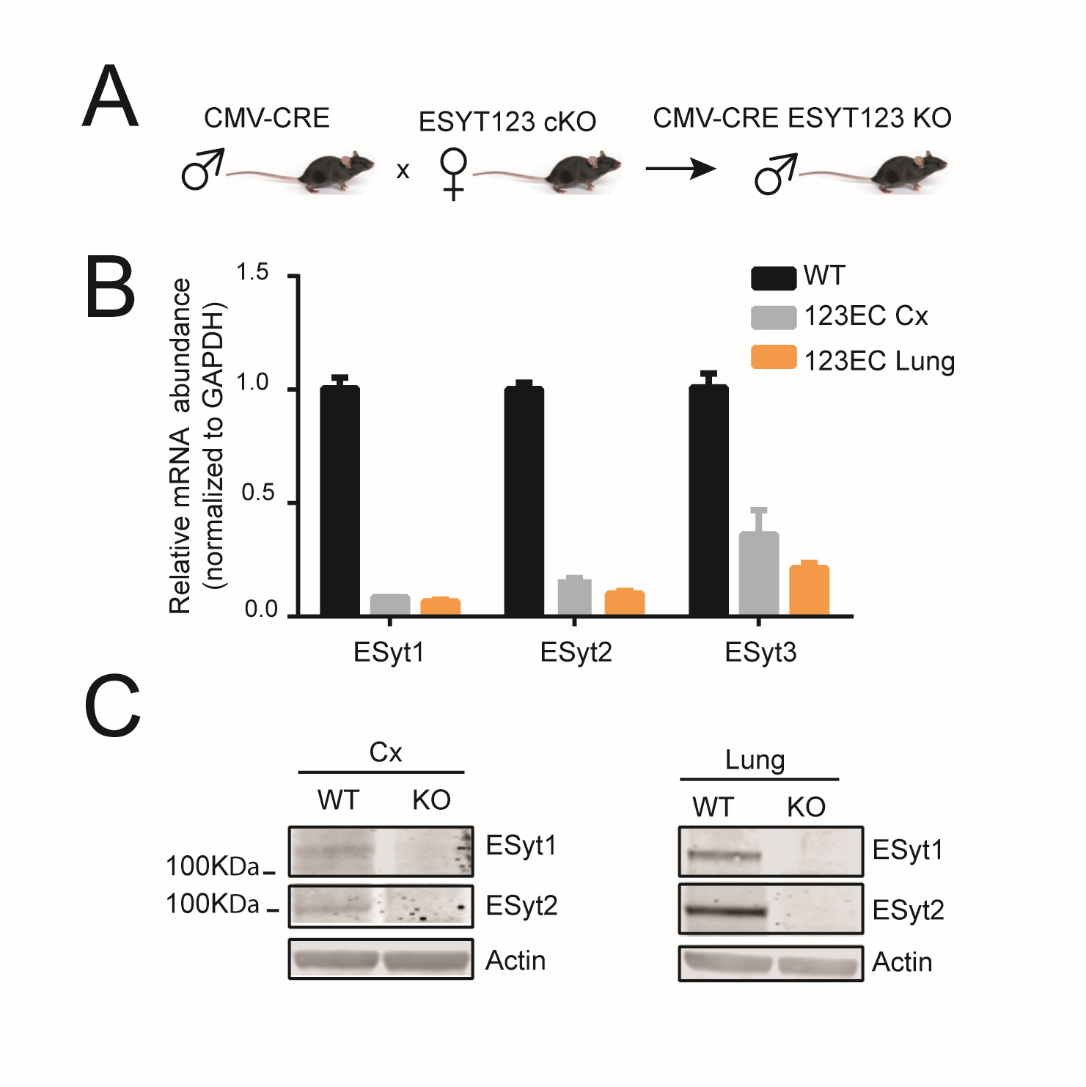


**S1 Fig.**

(A) Schematic of the breeding strategy used to obtain constitutive Esyt123 triple KO mice starting with the conditional KO mouse lines. ESyt123 triple cKO females were crossed with CMV-CRE males to generate constitutive Esyt123 triple KO mice after further interbreeding.

(B) RT-PCR measurements of ESyt1, ESyt2 and ESyt3 mRNA levels in the cortex and lung of WT and ESyt123 triple KO mice (123EC KO). Levels were normalized to GAPDH. Data are shown as means ± SEM, n=3. Note that mRNA measurements are not suitable for assessing the efficacy of a conditional KO since for many mRNAs, nonsense-mediated decay that destroys mRNAs containing a disrupted open reading frame either does not operate at all or is inefficient. Thus, many null alleles exhibit normal or partial mRNA levels that, however, do not encode a protein.

(C) Representative immunoblots for ESyt1 and ESyt2 showing that these proteins are not detectable in brain and lung samples from constitutive ESyt123 triple KO mice.

**SUPPLEMENTARY METHODS:**

**Sequences of the initial mutant alleles for ESyt1 and ESyt2**

Sequence of the Esyt1 mutant allele

**General design**: intron8 - EXON9 -intron9 - F3 – attP - LoxP2272 - intron9 - EXON10 - intron10 - LoxP2272 – attP – FRT - PGKpro-Neo-PGKpolyA - F3 - intron9 - EXON10(D4A) - intron10 – FRT - intron10 - EXON11 - intron11.

>EXON 9

ctttctctcaagggccctgtgctggggtcttagtttctcaccggtggattcctgaccccagGGCATTATCCGGATTCACC

TGCTGGCAGCCCGAGGTCTGAGTTCCAAGGACAAGTATGTGAAAGGCCTGATTGAGGGCAAATCGGATCCCTACGCGCTC

GTCCGTGTGGGCACCCAGACGTTCTGCAGCCGTGTCATAGATGAGGAGCTCAACCCTCACTGGGGAGAGACATATGAGgt

SphI

aggaagccaaggggcgtgtgtgcatgcgtgtgcatgtgtgtgtaccctcactggggagagatgtatgaggtaggaagccg

F3 attP

aggaagaagttcctattcTTCAAATAgtataggaacttctcaaggtcctacggagtaGTGCCCCAACTGGGGTAACCTtt

**LoxP2272**

gAGTTCTCTCAGTTGGGGGCGTAGGGTCtgctaatgataacttcgtataGGATACTTtatacgaagttatcatgaggccc

tgagaggcgatggtgggagaagctgctgactggatggcctgcatagctacagctcattaagaccacagtcctctgtgctc

>EXON 10 BssSI BamHI

tccagGTGATAGTCCACGAGGTTCCAGGACAGGAGATCGAGGTGGAGGTATTTGACAAGGATCCAGATAAAGATGATTTT

CTGGGAAGgtgagactgcccattttagattctaaagtcccaatgttaccaagattctggaagtagcagagtatgcacaca

caacacataccctccctttgtccctctgtccctccctcccctccatgtaaccactaagcgtgtactgctcttgtagagga

**LoxP2272 attP**

ccagagttcgatccctagcacataacttcgtataGGATACTTtatacgaagttatcgggagtaGTGCCCCAACTGGGGTA

FRT Neo

ACCTttgAGTTCTCTCAGTTGGGGGCGTAGGGTCgacatcgaGAAGTTCCTATTCTCTAGAAAGTATAGGAACTTCATGC

ATAAGGGTTCCGCAAGCTCTAGTCGAGCCCCAGCTGGTTCTTTCCGCCTCAGAAGCCATAGAGCCCACCGCATCCCCAGC

ATGCCTGCTATTGTCTTCCCAATCCTCCCCCTTGCTGTCCTGCCCCACCCCACCCCCCAGAATAGAATGACACCTACTCA

GACAATGCGATGCAATTTCCTCATTTTATTAGGAAAGGACAGTGGGAGTGGCACCTTCCAGGGTCAAGGAAGGCACGGGG

GAGGGGCAAACAACAGATGGCTGGCAACTAGAAGGCACAGTCGAGGCTGATCAGCGAGCTCTAGAGAATTGATCCCCTCA

GAAGAACTCGTCAAGAAGGCGATAGAAGGCGATGCGCTGCGAATCGGGAGCGGCGATACCGTAAAGCACGAGGAAGCGGT

CAGCCCATTCGCCGCCAAGCTCTTCAGCAATATCACGGGTAGCCAACGCTATGTCCTGATAGCGGTCCGCCACACCCAGC

CGGCCACAGTCGATGAATCCAGAAAAGCGGCCATTTTCCACCATGATATTCGGCAAGCAGGCATCGCCATGGGTCACGAC

GAGATCATCGCCGTCGGGCATGCGCGCCTTGAGCCTGGCGAACAGTTCGGCTGGCGCGAGCCCCTGATGCTCTTCGTCCA

GATCATCCTGATCGACAAGACCGGCTTCCATCCGAGTACGTGCTCGCTCGATGCGATGTTTCGCTTGGTGGTCGAATGGG

CAGGTAGCCGGATCAAGCGTATGCAGCCGCCGCATTGCATCAGCCATGATGGATACTTTCTCGGCAGGAGCAAGGTGAGA

TGACAGGAGATCCTGCCCCGGCACTTCGCCCAATAGCAGCCAGTCCCTTCCCGCTTCAGTGACAACGTCGAGCACAGCTG

CGCAAGGAACGCCCGTCGTGGCCAGCCACGATAGCCGCGCTGCCTCGTCCTGCAGTTCATTCAGGGCACCGGACAGGTCG

GTCTTGACAAAAAGAACCGGGCGCCCCTGCGCTGACAGCCGGAACACGGCGGCATCAGAGCAGCCGATTGTCTGTTGTGC

CCAGTCATAGCCGAATAGCCTCTCCACCCAAGCGGCCGGAGAACCTGCGTGCAATCCATCTTGTTCAATGGCCGATCCCA

TGGTTTAGTTCCTCACCTTGTCGTATTATACTATGCCGATATACTATGCCGATGATTAATTGTCAACAGGCTGCAGGTCG

AAAGGCCCGGAGATGAGGAAGAGGAGAACAGCGCGGCAGACGTGCGCTTTTGAAGCGTGCAGAATGCCGGGCCTCCGGAG

GACCTTCGGGCGCCCGCCCCGCCCCTGAGCCCGCCCCTGAGCCCGCCCCCGGACCCACCCCTTCCCAGCCTCTGAGCCCA

GAAAGCGAAGGAGCAAAGCTGCTATTGGCCGCTGCCCCAAAGGCCTACCCGCTTCCATTGCTCAGCGGTGCTGTCCATCT

GCACGAGACTAGTGAGACGTGCTACTTCCATTTGTCACGTCCTGCACGACGCGAGCTGCGGGGCGGGGGGGAACTTCCTG

ACTAGGGGAGGAGTAGAAGGTGGCGCGAAGGGGCCACCAAAGAACGGAGCCGGTTGGCGCCTACCGGTGGATGTGGAATG

TGTGCGAGGCCAGAGGCCACTTGTGTAGCGCCAAGTGCCCAGCGGGGCTGCTAAAGCGCATGCTCCAGACTGCCTTGGGA

F3

AAAGCGCCTCCCCTACCCGGTAGAATTTCGACGAACGCGTGAAGTTCCTATTCTTCAAATAGTATAGGAACTTCcatgag

gccctgagaggcgatggtgggagaagctgctgactggatggcctgcatagctacagctcattaagaccacagtcctctgt

EXON 10 (D4A)

gctctccagGTGATAGTCCACGAGGTTCCAGGACAGGAGATCGAGGTGGAGGTATTTGCCAAATCTCCAGATAAATCTGC

TTTCCTAGGAAGgtgagactgcccattttagattctaaagtcccaatgttaccaagattctggaagtagcagagtatgca

cacacaacacataccctccctttgtccctctgtccctccctcccctccatgtaaccactaagcgtgtactgctcttgtag

FRT

aggaccagagttcgatccctagcactgctaatgGAAGTTCCTATTCTCTAGAAAGTATAGGAACTTCgaggcttacaacc

BsrGI

tcctgtatctccagcttccatccctctggccttcttaggcacctgcactcacatgtacacatccacacacagaattttta

aaaataataaaataaaaaggattctccggttattcctggatggtgagtggagggaaacccgctgacttgctactaattat

gtccctctcctctcctctcctctcctctcctctcctctcctctcctctcctctcttcttctccccaccccaccccctctt

EXON 11

ccagAATGAAGCTGGACGTGGGGAAGGTATTACAGGCTGGAGTCCTGGATAATgtaagttgggagaagaggaagtgatct

cactctctgggaagtgacagctgctgccttctgggtggtctctgatctccgcttgtctccctttgactcccttcag

Sequence of the Esyt2 mutant allele

**General design**: EXON9 - intron9 - F3 - LoxP - intron9 - EXON10 - intron10 - LoxP - FRT - PGKpro-Neo-PGKpolyA - F3 - LoxP2272 - intron9 - EXON10(D4A) - intron10 - Lox2272 - FRT - intron10 - EXON11

EXON 9

tccattctccacagcaaacggtcagtggtttgttcctctcttcagGGTGTCCTAAGGATTCACTTTATTGAAGCTCAAGATCTTCAGGGCAAAGACACCTACCTTAAAGGCCTTGTCAAGGGAAAATCAGACCCCTATGGAATTATCCGGGTGGGCAACCAAATCTTCCAGAGCAAAGTCATCAAAGAGAACCTTAGTCCAAAATGGAATGAAGTGTATGAGgtaagtaagcagtcgggtcaaggtatggtaactgagagaggacacgagaaatctcattccagatctgtcgcacaaaagtgtggctaacttggaccattgttgaaggttttttaatttctcatttttgagacagtttgtactttctctcctaccacagaacaatcttgtaatgtgagcacataatagttttaagcaatggttacgtaaggctaccaaaatgatttgtggcaatatactttttaatgtaaataggttttctgattataaaatactatatgtggctaaaacacataaacatagaaatatgctgttttatacatttttatttaacatgtttggacaacttgcatctcagtggatagataatgggcattattggttttgttacattttgtttcccgttttgtgttttttctctctttctttttttaaaacattttatttccttcccttgagacggtgtttgtttattcatttatatcttgggatggcctatgactcctgatccttgtgcttcagcctcccaagcacttggtgctcactgccacacccagctaggatctacttgacttttgtagtcattccatt

NdeI

gaatttcttgacctggacaggcctgggccactaagtgcagtactataggtagtgctccttaagaataatgtcatatgtataaattgctggatccaaaactggaggttttcagaggacagaattgttgttgagaacaatggtgtgttctgctgttctgccccttctctgccaccctcagtcctctgtcacctggcagttcctattctctaagtggataagagtttttcaagatttgcctctgaa

F3

tggaaccagttcagaatttaggactattttctgtgtcttcacttg**GAAGTTCCTATTCTTCAAATAGTATAGGAACTTC**tc

LoxP

aaggtc**ATAACTTCGTATAGCATACATTATACGAAGTTAT**gtgtagaccttagattttatgtatgggtgacttcacactgccaccttgttttaggggcttgtgcttggcccactgacacagaattcggataaccagaaacaggcccagcagacttgttctgtgggcagatattttagaatggcctagtcatcaagaactgtatgtcagttttctgacagaaagaaagtagtatacttttaaaactgcttactgcaagagaaaagtacatggtaatatttgttatagtcatcatacttagcatttgattgtgatagtaaaactgc

EXON 10 Bam

ctaactcctaaccattgattttcagGCTTTAGTGTATGAACACCCTGGACAAGAATTGGAGATTGAACTGTTTGATGAGGA

HI

TCCAGACAAGGATGATTTCTTAGGAAGgtgaaaaaaagagcttgggaacatccttctaatcactctccttgatgcaaactttaagattcattcatttacacatacacattttaaatgccttttacataggtcatctattttaccacatatcttaatttttttttttacttctaaaaacactctgttgtccatcacagagacttacactgaggttcattccacctgtcagttagactatgcaag

LoxP FRT

ctggagagccagtgaatgcaga**ATAACTTCGTATAGCATACATTATACGAAGTTAT**acatcgaGAAGTTCCTATTCTCTAG

Neo

AAAGTATAGGAACTTCATGCATAAGGGTTCCGCAAGCTCTAGTCGAGCCCCAGCTGGTTCTTTCCGCCTCAGAAGCCATAGAGCCCACCGCATCCCCAGCATGCCTGCTATTGTCTTCCCAATCCTCCCCCTTGCTGTCCTGCCCCACCCCACCCCCCAGAATAGAATGACACCTACTCAGACAATGCGATGCAATTTCCTCATTTTATTAGGAAAGGACAGTGGGAGTGGCACCTTCCAGGGTCAAGGAAGGCACGGGGGAGGGGCAAACAACAGATGGCTGGCAACTAGAAGGCACAGTCGAGGCTGATCAGCGAGCTCTAGAGAATTGATCCCCTCAGAAGAACTCGTCAAGAAGGCGATAGAAGGCGATGCGCTGCGAATCGGGAGCGGCGATACCGTAAAGCACGAGGAAGCGGTCAGCCCATTCGCCGCCAAGCTCTTCAGCAATATCACGGGTAGCCAACGCTATGTCCTGATAGCGGTCCGCCACACCCAGCCGGCCACAGTCGATGAATCCAGAAAAGCGGCCATTTTCCACCATGATATTCGGCAAGCAGGCATCGCCATGGGTCACGACGAGATCATCGCCGTCGGGCATGCGCGCCTTGAGCCTGGCGAACAGTTCGGCTGGCGCGAGCCCCTGATGCTCTTCGTCCAGATCATCCTGATCGACAAGACCGGCTTCCATCCGAGTACGTGCTCGCTCGATGCGATGTTTCGCTTGGTGGTCGAATGGGCAGGTAGCCGGATCAAGCGTATGCAGCCGCCGCATTGCATCAGCCATGATGGATACTTTCTCGGCAGGAGCAAGGTGAGATGACAGGAGATCCTGCCCCGGCACTTCGCCCAATAGCAGCCAGTCCCTTCCCGCTTCAGTGACAACGTCGAGCACAGCTGCGCAAGGAACGCCCGTCGTGGCCAGCCACGATAGCCGCGCTGCCTCGTCCTGCAGTTCATTCAGGGCACCGGACAGGTCGGTCTTGACAAAAAGAACCGGGCGCCCCTGCGCTGACAGCCGGAACACGGCGGCATCAGAGCAGCCGATTGTCTGTTGTGCCCAGTCATAGCCGAATAGCCTCTCCACCCAAGCGGCCGGAGAACCTGCGTGCAATCCATCTTGTTCAATGGCCGATCCCATGGTTTAGTTCCTCACCTTGTCGTATTATACTATGCCGATATACTATGCCGATGATTAATTGTCAACAGGCTGCAGGTCGAAAGGCCCGGAGATGAGGAAGAGGAGAACAGCGCGGCAGACGTGCGCTTTTGAAGCGTGCAGAATGCCGGGCCTCCGGAGGACCTTCGGGCGCCCGCCCCGCCCCTGAGCCCGCCCCTGAGCCCGCCCCCGGACCCACCCCTTCCCAGCCTCTGAGCCCAGAAAGCGAAGGAGCAAAGCTGCTATTGGCCGCTGCCCCAAAGGCCTACCCGCTTCCATTGCTCAGCGGTGCTGTCCATCTGCACGAGACTAGTGAGACGTGCTACTTCCATTTGTCACGTCCTGCACGACGCGAGCTGCGGGGCGGGGGGGAACTTCCTGACTAGGGGAGGAGTAGAAGGTGGCGCGAAGGGGCCACCAAAGAACGGAGCCGGTTGGCGCCTACCGGTGGATGTGGAATG

TGTGCGAGGCCAGAGGCCACTTGTGTAGCGCCAAGTGCCCAGCGGGGCTGCTAAAGCGCATGCTCCAGACTGCCTTGGGAA

F3

AAGCGCCTCCCCTACCCGGTAGAATTTCGACGAACGCGTAAGTTCCTATTCTTCAAATAGTATAGGAACTTCtgctaatg**A**

**Lox2272**

TAACTTCGTATAGGATACTTTATACGAAGTTATgtgtagaccttagattttatgtatgggtgacttcacactgccaccttgttttaggggcttgtgcttggcccactgacacagaattcggataaccagaaacaggcccagcagacttgttctgtgggcagatattttagaatggcctagtcatcaagaactgtatgtcagttttctgacagaaagaaagtagtatacttttaaaactgcttactgcaagagaaaagtacatggtaatatttgttatagtcatcatacttagcatttgattgtgatagtaaaactgcctaactc

EXON 10 (D4A)

ctaaccattgattttcagGCTTTAGTGTATGAACACCCTGGACAAGAATTGGAGATTGAgCTcTTTGcTGAAtcTCCAGACAAGtcTGcTTTCTTAGGAAGgtgaaaaaaagagcttgggaacatccttctaatcactctccttgatgcaaactttaagattcattcatttacacatacacattttaaatgccttttacataggtcatctattttaccacatatcttaatttttttttttacttctaaaaacactctgttgtccatcacagagacttacactgaggttcattccacctgtcagttagactatgcaagctggaga

Lox2272 FRT

gccagtgaatgcagaATAACTTCGTATAGGATACTTTATACGAAGTTATgtcagtcGAAGTTCCTATTCTCTAGAAAGTATAGGAACTTCgctccctcacccccagccccactttgtgcagggaagtctgaaactacttccttccaggttgtcaatgtggacttaatgttaagcttttcagtttgctagggtacagtcactttcatgatgcccacatattgggatggtaaataatgtttactccagttgaagaactaatatacctggggaaatgggcttttaaaatatattcctgactgacagatggctcagtggttaagaatgcttgcttcccttcctctgtacctaacacctttgtcagatggctcacagctgcctaaaactctagctcctctggctctgtgggtacctgcattcacctgggttttgtttttttgtttgtttgtttgtttgtttgttttttgtttttcatgcacacacatattaagatagcaaaaataaatattaaaaaatatgtttccaggactgagaagatagctcagttggaatgattctcatgattcataccagtagcaaaatcacagttatgaaaattgttacaatcatcatactgtaatctccattactgtgaactctttacccatctctgccccagtgccaccagtgcatcacagtgtgtccacaataaaggcagtctgttattctatgctttttacatcttgtgagagatggctcagtggctaagaatacttcttcccaagttcggtgcgtagcacccatgtcagatggctcatggctgtctgttatattagttcttcagctgtaataaacaaatcagtatttactgttccaatacatgggtacatctcaggcctccacatatgtggtaaaataaatgtcatacagcaataaaagtacttttatggttgggggtcaccacaacgtgaagaactatattaaagggtcgcagcattaggaaggctgagaaccactggcttagtttttagaatgctttcattgaaagcataagagaccattgatggatgggtggatagatagatagatagatagatagatagatagatagatagatagaaagaaagaaagaaagaaagaaagaaagaaagaaagaaagaaagaaagaaagaaagaaagaaagaaagaaaggaagaaaccacatggtggctcacaaccatccaggtaatgggatccaatgccttcttctggtgtatatgaaaacagctgcagtgtacttgtaaataaaataaatctttaaaaaaaaaagggtggggggttgagcatagtgacacaggcttgtaatctctggggatggattgcactggggaagcaaagacaagtatatccttggggctaattaggggcaactaaagtacttggtaagtttcattctaggtcagtgagataccgtgtttcaagaaaacaaaaactgtacataaagaaaaacattggcctccacagatttgtatttgcatctgtacacaaaacgaataaatatgcatgcacacatttaatacatgtttatctaattaaggttatttaaatgagtttcttgtggagagggatggtgaaggattgggttacattaccaaggattatgcctcatcaagtatggccttgtgttttatgctctgacttgagagtctctaagacctatggattgtactgtgttttcagaatctgtgattgcatacaagcttaggagcaggaacgtggtccaatcaaatatgtatataagatgagaggctggtaaagcagctaggagcatcttgtcattgactagggctaagactggactgattcaccctgaaacccagattgtaagggagaagggagatatgttcaaaagtacaagtatactaatcccttcatcatcattgctggctccagagtggtagtgccatgattgaattagagtaggattgtttggtggtaatagaacctactctagactgctaagggacttgatatgatgtggacttacacttcattatacaagtaactgtc

NheI

cacagtgatgactgagcacaaaaggataacatgtaaaaactcatcagctagccaagcatggtggtgtacacctttaatcccagtacttgggaagcagaggcaggcaggtctcttgagttaaaagccagcctcgtctatattatgagaccctgtctaagaaacagaaaatcacctgtggctggggaaatcagtgcatgaagaacttgccatgtaagcataggatcaaagttcagacccagaacccacataaatgccagaaaaacaacacagctgcctgtaattctagcaattggaaggcagaggctgatatggccagaacagttctactatttaaactagtcatatcaggctctgggttctactggaagactgtcataatgagtaagatgaagagtgtttgaggaagactcctgaggtttgtctcaggcgtccacgtacgagaatgctgtcagttcccattgctggtcgtaggctttcacgtgcatgtccacatgagcagtctgttcctgatgtttatctcaggcctccatgtgcacgcacacatgagcagtcagttctagcctatgtgttctagtgttagttctagtatatgtgttcagtattttgaaaattaatcctgttatttcttactcttgattttattgggcataagttgtaagaagcattgaggaaagtttcctggtaaattgtatagctttctttttcttttttctttttttgcatttctagagtactttttcacagacaatgctcagcagttcagaagtgcatttctgtgttttaaaattttgtgttgttttattcttacag

EXON 11

TCTTATGATTGATCTTATTGAAGTTGAGAAGGAGCGCCTTTTAGATGAA
