# Supplementary figures and images for: Correction: Extended Synaptotagmin (ESyt) Triple Knock-Out Mice Are Viable and Fertile without Obvious Endoplasmic Reticulum Dysfunction
Source: PLoS One. 2024 Feb 6;19(2):e0298645. doi: 10.1371/journal.pone.0298645 (PMC10846706; doi:10.1371/journal.pone.0298645)

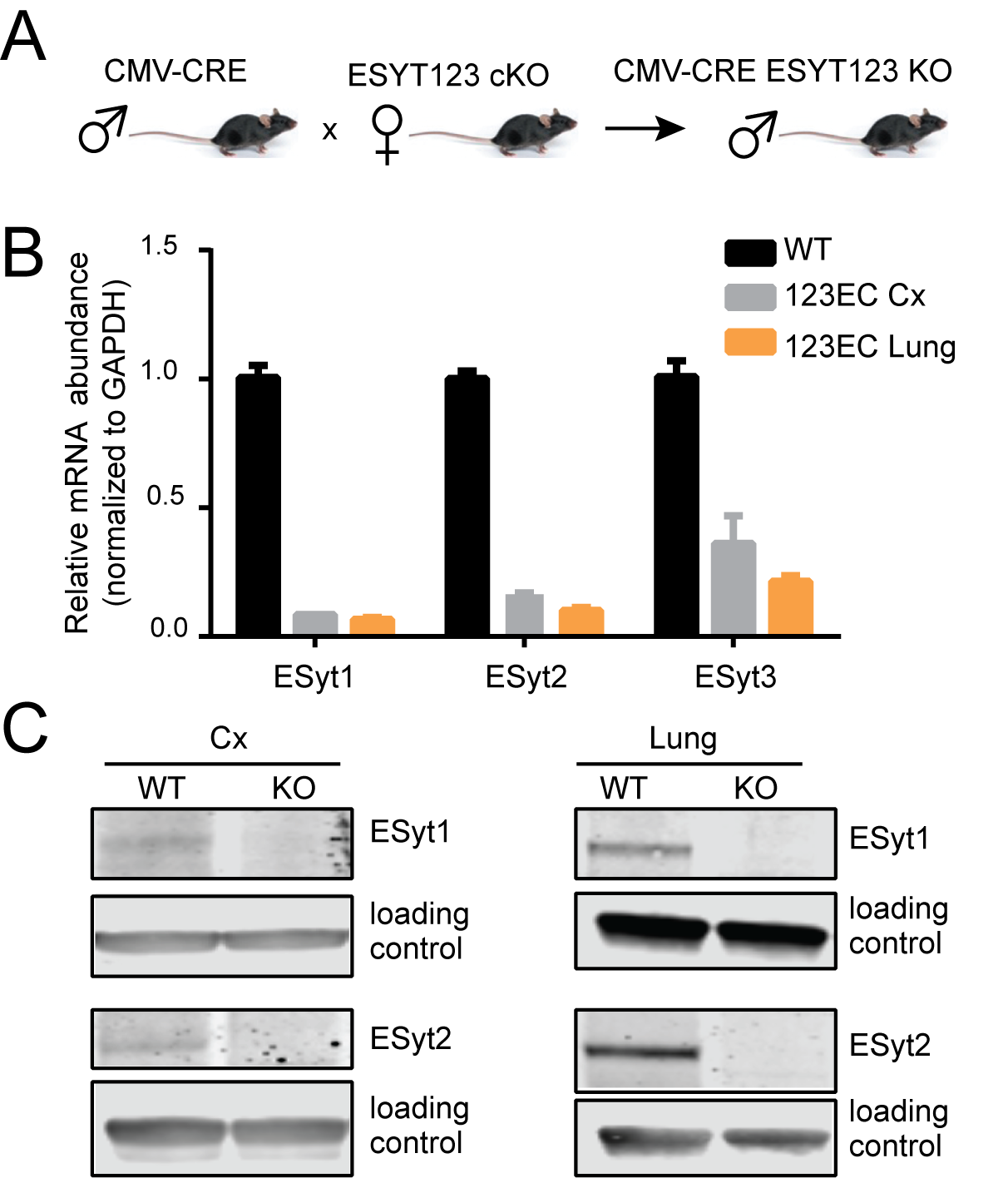

Supplement: S1 Fig — (A) Schematic of the breeding strategy used to obtain constitutive Esyt123 triple KO mice starting with the conditional KO mouse lines. Esyt123 triple cKO females were crossed with CMV-CRE males to generate constitutive Esyt123 triple KO mice after further interbreeding. (B) RT-PCR measurements of Esyt1, Esyt2 and Esyt3 mRNA levels in the cortex and lung of WT and Esyt123 triple KO mice (123EC KO). Levels were normalized to GAPDH. Data are shown as means ± SEM, n = 3. Note that mRNA measurements are not suitable for assessing the efficacy of a conditional KO since for many mRNAs, nonsense-mediated decay that destroys mRNAs containing a disrupted open reading frame either does not operate at all or is inefficient. Thus, many null alleles exhibit normal or partial mRNA levels that, however, do not encode a protein. (C) Representative immunoblots for Esyt1 and Esyt2 showing that these proteins are not detectable in brain and lung samples from constitutive Esyt123 triple KO mice (loading controls = actin). (TIF) [file pone.0298645.s001.tif]

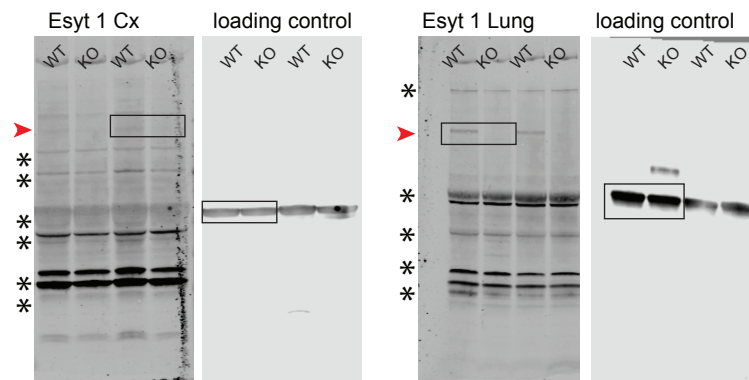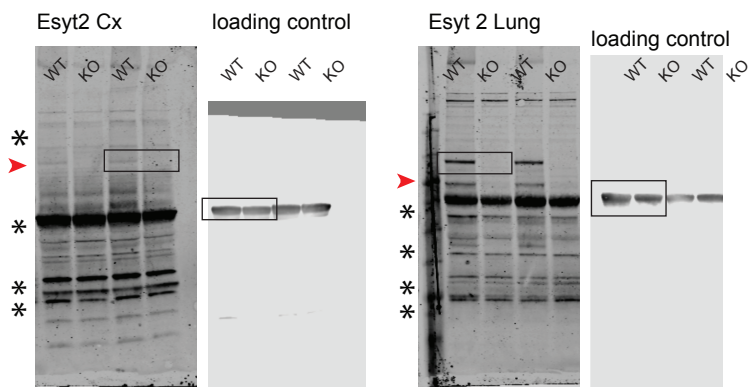

Supplement: S3 File — Annotated and individual unannotated images underlying western blots. (PDF) [file pone.0298645.s004.pdf]
