# Supplementary material for: Correction: Extended Synaptotagmin (ESyt) Triple Knock-Out Mice Are Viable and Fertile without Obvious Endoplasmic Reticulum Dysfunction
Source: PLoS One. 2024 Feb 6;19(2):e0298645. doi: 10.1371/journal.pone.0298645 (PMC10846706; doi:10.1371/journal.pone.0298645)
Supplement: S1 File — Annotated and individual unannotated images underlying all western blots in this figure panel. (PDF) [file pone.0298645.s002.pdf]

Black-and-white view of green & red fluorescent signals for Fig. 3A

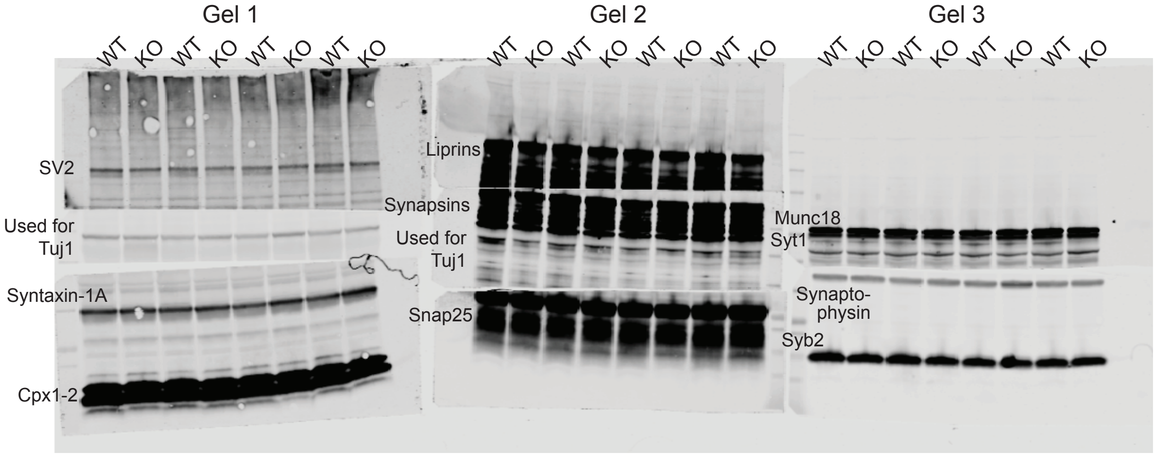

Color views of green & red fluorescent signals for Fig. 3A
(for gel 2, the red fluorescence is only shown in the second panel)

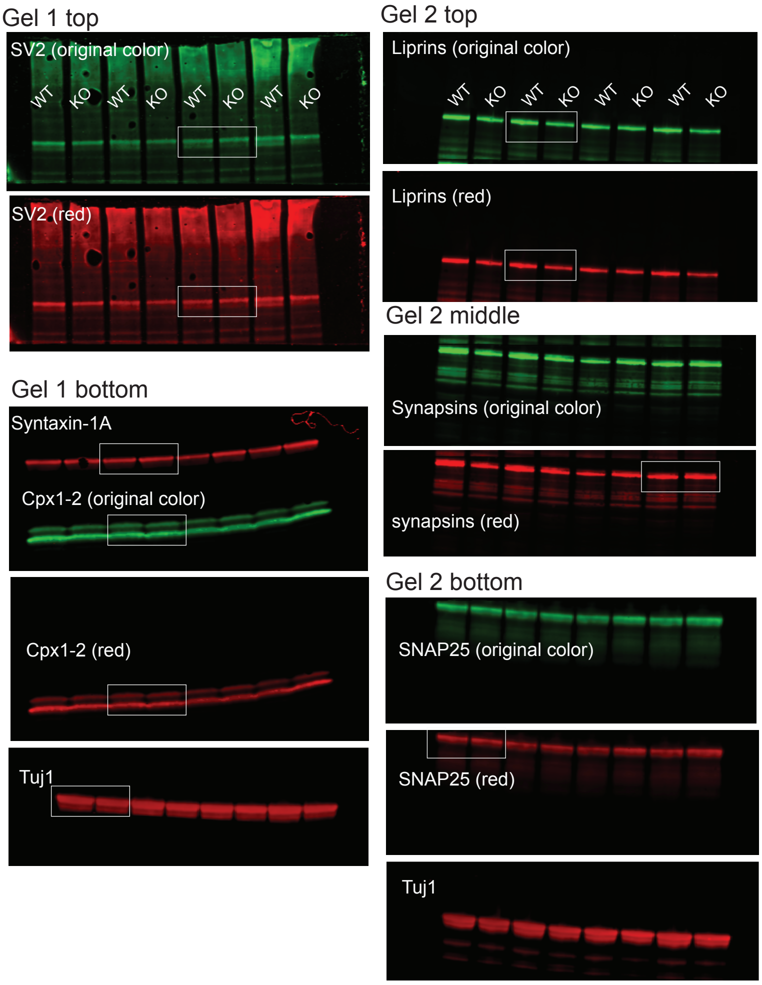

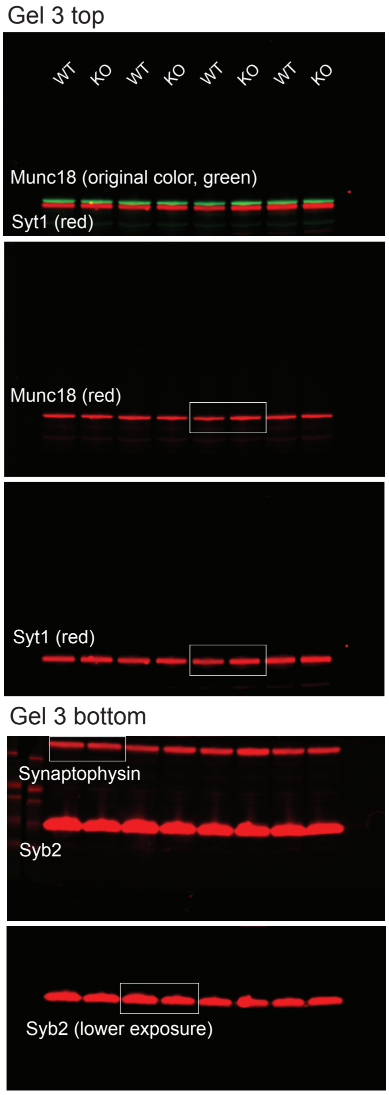

Note: Complexin-1 & -2 exhibit differential abudance, as do the four principal synapsins (Synapsin-1A, -1B, -2A, and -2B). Munc18 & Syt1 have similar sizes but different secondary antibodies.Middle stripes from gel1 and gel2 were stained for Tuj1 after the firstsynaptic stainings were acquired, therefore are not present in the gel overview shown on top
