# Supplementary material for: Correction: Extended Synaptotagmin (ESyt) Triple Knock-Out Mice Are Viable and Fertile without Obvious Endoplasmic Reticulum Dysfunction
Source: PLoS One. 2024 Feb 6;19(2):e0298645. doi: 10.1371/journal.pone.0298645 (PMC10846706; doi:10.1371/journal.pone.0298645)
Supplement: S2 File — Annotated and individual unannotated images underlying all western blots in this figure panel except for Nir2. The white box is incorrectly positioned on the Gpr78 image: lanes 5–6 are shown in the figure. (PDF) [file pone.0298645.s003.pdf]

For Figure 3C, the blots were probed with antibodies that were reacted with green or red secondary fluorescent antibodies depending on the species of the primary antibody. Some of the proteins were probed on the same gels used for Figure 3A (HSP70 on Gel 1 and Gel2, HSP27 on Gel 3). In addition, VAP-a, VAP-b and Gpr78 were run on two gels in parallel with the same samples used in Figure 3A. Tuj1 controls were included for gel4, while Tuj1 controls from gel1 and 2 (see Figure 3A) were used to normalized HSP70 and HSP27 signals.

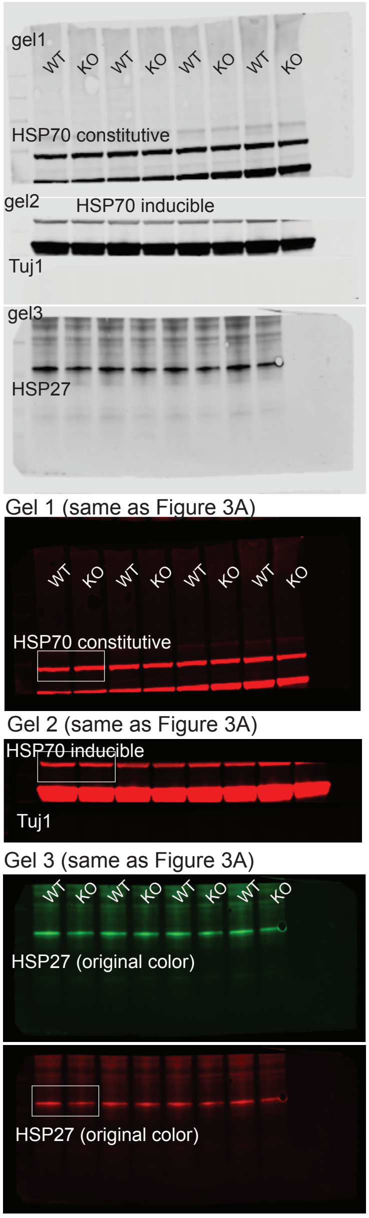

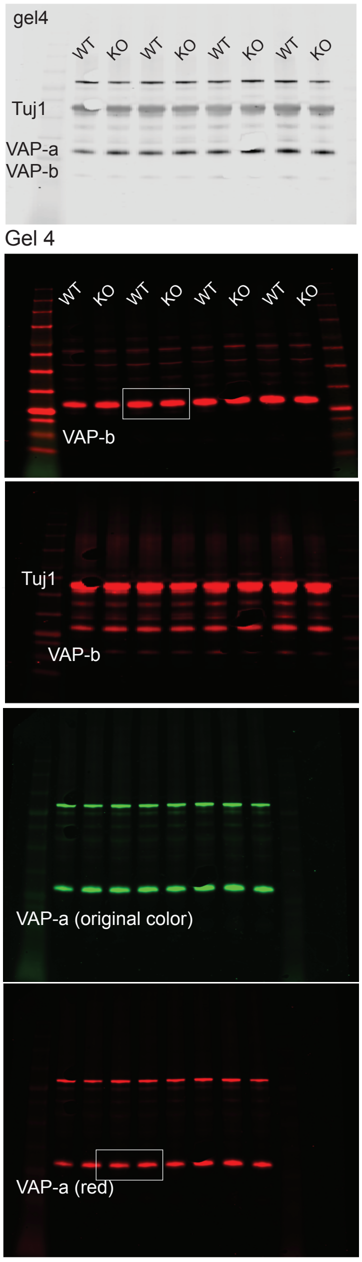

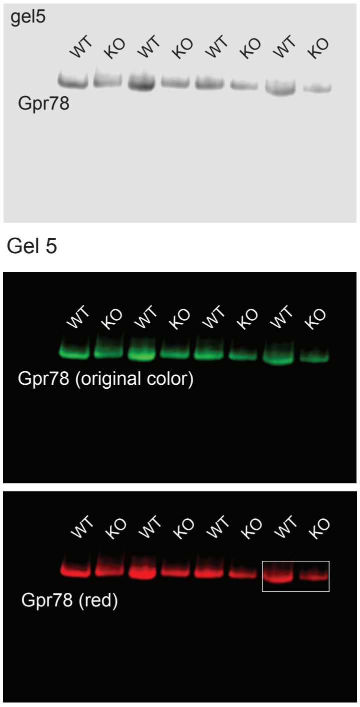

Note: For gel 4, all stainings were done sequentially. VAP-b signal was acquired first (immuno-stained with a specific VAP-b mouse antibody, in red), followed by Tuj1 signal (mouse antibody, in red) and VAP-a (rabbit antibody in green). VAP-b and VAP-b have similar sizes but different secondary antibodies.

Note: HSP27 and HSP70 were run on the same membranes used for figure 3A.
